# Evaluation of the antibacterial activity of *Elsholtzia ciliate* essential oil against halitosis-related *Fusobacterium nucleatum* and *Porphyromonas gingivalis*

**DOI:** 10.3389/fmicb.2023.1219004

**Published:** 2023-08-07

**Authors:** Fengjiao Li, Chuandong Wang, Jing Xu, Xiaoyu Wang, Meng Cao, Shuhua Wang, Tingting Zhang, Yanyong Xu, Jing Wang, Shaobin Pan, Wei Hu

**Affiliations:** ^1^College of Pharmacy, Shandong University of Traditional Chinese Medicine, Jinan, China; ^2^State Key Laboratory of Microbial Technology, Microbial Technology Institute, Shandong University, Qingdao, China; ^3^Shenzhen RELX Technology Co., Ltd., Shenzhen, China; ^4^Shandong Aobo Biotechnology Co., Ltd., Liaocheng, China; ^5^Beijing Xinyue Technology Co., Ltd., Beijing, China

**Keywords:** *Elsholtzia ciliate*, essential oil, halitosis, *Fusobacterium nucleatum*, *Porphyromonas gingivalis*, antibacterial activity, biofilm

## Abstract

The broad-spectrum antimicrobial activity of *Elsholtzia ciliate* essential oil (ECO) has been previously reported, but its effectiveness against halitosis-causing bacteria such as *Fusobacterium nucleatum* and *Porphyromonas gingivalis* is not well understood. In this study, we investigated the bacteriostatic activity of ECO against planktonic cells and biofilms of *F. nucleatum* and *P. gingivalis*, as well as its ability to inhibit bacterial metabolism and production of volatile sulfur compounds (VSCs) at sub-lethal concentrations. Our findings revealed that ECO exhibited comparable activities to chlorhexidine against these oral bacteria. Treatment with ECO significantly reduced the production of VSCs, including hydrogen sulfide, dimethyl disulfide, and methanethiol, which are major contributors to bad breath. As the major chemical components of ECO, carvacrol, p-cymene, and phellandrene, were demonstrated *in vitro* inhibitory effects on *F. nucleatum* and *P. gingivalis*, and their combined use showed synergistic and additive effects, suggesting that the overall activity of ECO is derived from the cumulative or synergistic effect of multiple active components. ECO was found to have a destructive effect on the bacterial cell membrane by examining the cell morphology and permeability. Furthermore, the application of ECO induced significant changes in the bacterial composition of saliva-derived biofilm, resulting in the elimination of bacterial species that contribute to halitosis, including *Fusobacterium*, *Porphyromonas*, and *Prevotella*. These results provide experimental evidence for the potential clinical applications of ECOs in the prevention and treatment of halitosis.

## Introduction

Halitosis, also known as bad breath, is the third most common oral disease in clinical practice, with a high prevalence rate of up to 50% (Sombie et al., 2018). Halitosis originating from the mouth is mainly due to the overgrowth of some Gram-negative bacteria, e.g., *Fusobacterium nucleatum* and *Porphyromonas gingivalis*, which can cause biofilm-related infections such as dental plaque, oral ulcers, and periodontitis, and produce volatile sulfur compounds (VSCs), such as hydrogen sulfide, methyl mercaptan, and dimethylamine, resulting in malodorous gases (Nakhleh et al., 2018; Wu et al., 2020). *F. nucleatum* has a certain adhesion ability to oral epithelial cells and enamel, and is a critical participant in oral biofilm formation (Liu J. et al., 2013; Xu et al., 2020). It produces cysteine desulphurizing enzyme as its most important virulence factor to degrade L-cysteine and produce hydrogen sulfide (Chen, Y. et al., 2022). *P. gingivalis* is also involved in oral biofilm formation and its gingival protease plays an important role in the pathogenesis of periodontitis and degrades amino acids to produce VSCs, leading to bad breath. Its lipopolysaccharide component also causes gingivitis that exacerbates halitosis (Seerangaiyan et al., 2018; Mogilnicka et al., 2020).

Targeted inhibition and removal of pathogenic bacteria that produce VSCs in the oral cavity are currently one of the most effective methods for treating halitosis (Chen et al., 2010; Ortiz and Filippi, 2021). In addition to mechanical therapy, there are many forms of chemotherapeutic treatment that might be recommended by the dentist (Wu et al., 2020). Chlorhexidine (CHX), hexadecyl pyridinium chloride, and triclosan are effective compounds in reducing bad breath due to their broad-spectrum bactericidal activity. Currently, toothpaste and mouthwash on the market normally contain these antimicrobial compounds, which can significantly change the oral microbial community and cause adverse side effects such as vomiting, diarrhea, and microbial dysbiosis (Kumbargere Nagraj et al., 2019; Solderer et al., 2019). CHX is an important antibacterial agent that can inhibit a variety of microorganisms, control the formation of dental plaque, and thus reduce bad breath. Its mode of action is to penetrate the bacterial cell membrane, causing cell leakage and interruption of metabolism, thereby inhibiting cell growth (Cieplik et al., 2019; Ma et al., 2023). However, this treatment has some side effects prompting usage risks, such as causing taste abnormalities and eversible staining of teeth (Poppolo Deus and Ouanounou, 2022). Meanwhile, the success of peppermint mouth rinses as safe formulations to improve halitosis measures (Haghgoo and Abbasi, 2013) suggests that essential oils derived from medicinal plants may have good prospects for application in the treatment of bad breath. As natural broad-spectrum antibacterial agents, plant essential oils have the advantages of diverse composition, comprehensive sources, and low prices (Dobler et al., 2020), and now become a hot topic in antibacterial drug research due to their high efficiency, safety, low toxicity, and high stability (Tariq et al., 2019; Dobler et al., 2020; Tosun et al., 2022).

*Elsholtzia ciliata* (Thunb.) is an annual herb of the *Lamiaceae* family with a slightly spicy taste. In the practice of traditional Chinese medicine, it is commonly used for heatstroke, chest tightness, halitosis, acute gastroenteritis, etc. (Hou and Jiang, 2013; Wang et al., 2022). *E. ciliata* contains many volatile essential oils to produce a strong aroma, of which the main components are terpenoids and phenolic compounds, with the highest content being monoterpenoids (Pudziuvelyte et al., 2020). *E. ciliata* essential oil shows a broad-spectrum antimicrobial activity against *Escherichia coli*, *Pseudomonas aeruginosa*, *Salmonella typhi*, *Streptococcus mutans*, *Staphylococcus aureus*, and *Lactobacillus* spp. (Wang et al., 2022). Up to date, there is limited research on the inhibitory and bactericidal activities of *E. ciliata* essential oil against bad breath-related bacteria, with only a small amount of research focusing on the optimization of its extraction process and the elucidation of its chemical compositions (Pudziuvelyte et al., 2020). Therefore, using *E. ciliata* essential oil as a major object in the current study, we sought to systematically evaluate its *in vitro* antibacterial activity against halitosis-related bacteria, i.e., *F. nucleatum* and *P. gingivalis*, and their biofilms, inhibition of bacterial metabolic process and VSCs production, possible mechanisms of action, and effects on the bacterial populations within the saliva-derived biofilm, providing some experimental evidence for its potential applications in the clinical prevention and treatment of halitosis.

## Materials and methods

### Bacterial strains and plant materials

*Fusobacterium nucleatum* ATCC 25586 and *P. gingivalis* ATCC 33277 were both obtained from the Industrial Microbial Culture Collection and Management Center of China, and routinely cultivated on brain heart infusion (BHI, Difco, MD, United States) medium containing 0.005% hemin chloride and 0.001% vitamin K. The origin of the *Elsholtzia ciliata* (Thunb.) plant is from Yichun (Jiangxi, China; geographic coordinates are 113°54′–116°27′ E, 27°33′–29°06′N). *E. ciliate* essential oil (abbreviated as ECO in the text) was prepared from the stems and leaves of *E. ciliate* using a steam distillation method as previously described (Aziz et al., 2018).

### Determination of the antibacterial activity against bacterial planktonic cells

1 × 10^8^ cells/mL *F. nucleatum* and the 2 × 10^8^ cells/mL *P. gingivalis* diluted by BHI broth were, respectively, added to a 96-well tissue culture plate (Orange Scientific, BA, Belgium) with different concentrations of samples for testing. The bacterial cell concentrations were measured by reading OD_600 nm_ on a plate spectrophotometer (EZ Read 800 Plus, Biochrom, Cambridge, United Kingdom). The minimum inhibitory concentration (MIC) and minimum bactericidal concentration (MBC) were determined as previously described (Chitsazha et al., 2022).

### Determination of VSCs inhibition by Halimeter

*F. nucleatum* (1 × 10^8^ cells/mL) or *P. gingivalis* (2 × 10^8^ cells/mL) was inoculated in BHI broth, and a 5 mL aliquot was cultivated in 10 mL sterile glass tubes using an anaerobic incubator (Nuoxuan, Shanghai, China) for 12 h, and the tubes were shaking vigorously during the incubation to prevent biofilms formation. CHX or ECO was added to the broth at the concentration of 1/2× MIC. After incubation, a Halimeter (Yuanxun, Guangdong, China) was employed to measure VSCs concentration and record the data according to a published protocol (Tanabe et al., 2012). The inhibition rate of VSCs production by CHX or ECO was calculated as the percentage reduction in VSCs production of the treated group compared to the untreated control group. Triplicate experiments were conducted.

### Bacterial growth curve

*Fusobacterium nucleatum* (1 × 10^8^ cells/mL) or *P. gingivalis* (2 × 10^8^ cells/mL) was inoculated in BHI broth in a 24-well tissue culture plate (Orange Scientific) with different concentrations of CHX or ECO, incubated at 37°C for 12 h, and the plates were shaking vigorously during the incubation to prevent biofilms formation. The bacterial cell concentrations were measured by reading OD_600 nm_ at different time points to generate the growth curve.

### Bacterial biofilm elimination and metabolic inhibition

Bacterial biofilms were cultivated and quantified as previously described (Aherne et al., 2022) with minor modifications. *Fusobacterium nucleatum* and *P. gingivalis* cells were mixed in BHI broth at a final concentration of 1 × 10^8^ cells/mL and 2 × 10^8^ cells/mL, respectively, and added to a 96-well plate for anaerobic incubation for 48 h. Remove the supernatant, refill the well with fresh BHI broth containing different amounts of ECO, and anaerobically incubated for 24 h. Remove the supernatant, stain the biofilms with 0.1% crystal violet, and wash with sterile PBS three times, add 200 μL of 33% acetic acid to the well. The biomass of biofilm was determined by measuring OD_590 nm_ on a plate spectrophotometer. The biofilm elimination rate of ECO was calculated as the percentage reduction in biomass between the treated group and the untreated group. Triplicate experiments were conducted.

The 3-(4,5-dimethylthiazol-2-yl)-2,5-diphenyl tetrazolium bromide (MTT, Solarbio, United States) colorimetric assay (He et al., 2022) was employed to determine the metabolic activity of biofilms. Briefly, the 48 h-biofilms were prepared as described above, after removing the supernatant, the biofilms were washed three times with sterile PBS, and 200 μL PBS containing different amounts of ECO was refilled and incubated anaerobically for 4 h. 90 μL BHI and 10 μL of 5 mg/mL MTT were added to each well, and incubated at 37°C in the dark for 2 h. After incubation, discard the supernatant and wash the well with PBS, and add 200 μL DMSO to each well. The OD_490 nm_ was read on a plate spectrophotometer. The biofilm metabolic inhibition rate was calculated as the percentage reduction in OD_490 nm_ value between the treated group and the untreated group. Triplicate experiments were conducted.

### Confocal laser scanning microscopy

The biofilm formed by *Fusobacterium nucleatum* and *P. gingivalis* was cultured in a confocal laser petri dish (Solarbio, Beijing, China) as described above. After 48 h incubation, discard the supernatant and wash the well with PBS, and add PBS containing ECO or CHX at a concentration of 4× MIC, respectively. After incubation at 37°C for 4 h, the biofilms were stained SYTO 9 and propidium iodide (PI) within the LIVE/DEAD viability kit (Thermo Fisher, MA, United States) following the producer’s instruction. An LSM880 confocal laser scanning microscopy (CLSM) (Zeiss, Oberkochen, Germany) was used to observe and photograph the data through a 40× objective lens. The CLSM images were exported in an 8-bit TIFF format and statistically analyzed using the ImageJ software (Memmel et al., 2020). At least three random fields of each biofilm sample were examined.

### Gas chromatography-mass spectrometry analysis

The Q Exactive GC Orbitrap GC-MS/MS system equipped with a DB-5 MS U1tra Inert weakly polar column (30 m, 0.25 mm, 0.25 μm; Agilent, Santa Clara, CA, United States) was utilized for gas chromatography-mass spectrometry (GC-MS) analysis. The electron ionization (EI) source was employed and the interface temperature was set at 230°C. The full scan mode was utilized for data acquisition with the *m*/*z* range of 40–500 amu. The incubation temperature was 37°C with a 30 min incubation time, carrier gas flow rate was 1 mL/min, and injection temperature was 200°C. The temperature program for the GC analysis was as follows: started at 35°C for 6 min, ramped to 150°C at 4°C/min and held for 3 min, further ramped to 250°C at 40°C/min and held for 5 min, resulting in a total runtime of 45 min. The relative percent content of each component was determined by normalizing the peak areas of the TIC chromatogram. The mass spectrum was utilized to identify the structures of the components by searching NIST20 and WILEY275 databases and comparing with authentic standard spectra. The main chemical components of BCO were determined and quantified using direct liquid injection mode.

For biofilm VSCs analysis, 5 mL of BHI broth containing a concentration of 1 × 10^8^ cells/mL *F. nucleatum* or 2 × 10^8^ cells/mL *P. gingivalis* was added to sterilized headspace injection vials, respectively. The vials were then anaerobically incubated at 37°C for 12 h. In the drug treatment group, BCO was added at a final concentration of 2× MIC, whereas no drugs were added in the control group, and BHI medium without bacterial cells was used as a negative control. Gas composition in the headspace of the vials was analyzed using GC-MS with headspace injection mode (Milanowski et al., 2019), and the parameters are the same as described above.

### Antibacterial activity of the major chemical components in ECO

According to the GC-MS results, carvacrol, p-cymene, and phellandrene were identified as the major chemical components in ECO, therefore, the standard compounds of carvacrol, p-cymene and phellandrene were obtained from Macklin Biochemical Co. (Shanghai, China) for further experiments. MIC and MBC of the compounds against *F. nucleatum* and *P. gingivalis* planktonic cells were determined as mentioned above. Furthermore, using a checkerboard assay, the antibacterial effect of compound combinations was evaluated by performing concentration gradient joint sensitivity experiments as previously described (Priya et al., 2021). The judgment criterion for the sensitivity experiment of combination drug therapy is based on the fractional inhibitory concentration index (FICI). FICI ≤0.5 indicates synergistic effect, 0.5 < FICI ≤ 1.0 indicates additive effect, 1 < FICI ≤ 2 indicates irrelevant effect, and FICI>2 indicates antagonistic effect.

### Detection of hydrogen sulfide and methanethiol in VSCs

The cysteine stimulation test and methanethiol stimulation test were used to detect hydrogen sulfide and methanethiol in VSCs according to a published protocol (Liu P. F. et al., 2013) with minor modifications. To determine the concentration of hydrogen sulfide, the experimental setup was the same as that of MIC determination, and 10 μL of 0.6% cysteine solution and 10 μL of 2.4% lead acetate solution were added to each well. The plate was incubated anaerobically at 37°C for 12 h, the tubes were shaking vigorously during the incubation to prevent biofilm formation, and OD_550 nm_ was measured on a plate spectrophotometer. To determine the concentration of hydrogen sulfide, 10 μL of 1.8% methionine solution and 10 μL of 0.18% DTNB solution were added to each well, and OD_430 nm_ was measured. Triplicate experiments were conducted. The VSCs production rate was calculated as the percentage of hydrogen sulfide and methanethiol production in the treated group versus that of the untreated group. Triplicate experiments were conducted.

### Scanning electron microscopy

*F. nucleatum* and *P. gingivalis* cells were harvested, washed with PBS buffer for three times, resuspended in PBS with or without 2× MIC ECO, and incubated at room temperature for 4 h. The cells were collected by centrifugation and fixed with 2.5% glutaraldehyde (Thermo Fisher) for 3 h, and subsequently dehydrated using different concentrations of ethanol. The specimen was observed through a scanning electron microscope (Inspect F50, FEI, United States).

### Determination of cell membrane permeability

*F. nucleatum* and *P. gingivalis* cells were collected by centrifugation, resuspended in PBS with or without 2× MIC ECO or CHX to a final concentration of 1 × 10^8^ cells/mL and 2 × 10^8^ cells/mL, and incubated in anaerobic incubator for 10 h. For every 2 h, a small aliquot was taken and stained with PI for 15 min under dark conditions. The fluorescence intensity was measured using a fluorescence microplate reader (TECAN, Shanghai, China), with excitation at 490 nm and emission at 635 nm. Triplicate experiments were conducted.

### Cultivation of the saliva-derived bacterial biofilm

The saliva-derived bacterial biofilms containing halitosis-causing bacteria were cultivated according to a published method (Xu et al., 2022) with modifications. Ten healthy volunteers from Shandong University were selected, who had not taken any proton pump inhibitors, antibiotics, or corticosteroid medications, and had no other oral diseases, such as dental caries, periodontitis, or oral ulcers. Pooled saliva samples were collected immediately after waking up in the morning, added to a BHI medium, and incubated under microaerobic conditions at 37°C for 12 h. *F. nucleatum* and *P. gingivalis* were added in the broth at a final concentration of 5 × 10^2^ cells/mL and 1 × 10^3^ cells/mL, respectively. The broth was further incubated anaerobically at 37°C for 36 h to obtain the saliva-derived bacterial biofilm.

For the treatment experiment, CHX or ECO was added to the saliva-derived bacterial biofilm after 36 h incubation, e.g., 24 h after the addition of *F. nucleatum* and *P. gingivalis*, to a final concentration of 1× MIC, and BHI liquid was added to the control group. After cultivation for additional 12 h, the bacterial cells were collected and stored at −80°C before further testing.

### Microbial diversity analysis of the saliva-derived bacterial biofilm

Total genomic DNA was extracted from nine biofilm samples using the TGuide S96 Kit (Tiangen, Beijing, China) according to the manufacturer’s instructions. The hypervariable region V3-V4 of the bacterial 16S rRNA gene was amplified with primer pairs 338F: 5′-ACTCCTACGGGAGGCAGCA-3′ and 806R: 5′-GGACTACHVGGGTWTCTAAT-3′. PCR products were checked on agarose gel and purified through the Omega DNA purification kit (Omega, GA, United States). The purified PCR products were collected and the paired ends (2 × 250 bp) were performed on the Illumina Novaseq 6000 platform in Biomarker Technologies Co. (Beijing, China).

The qualified sequences with more than 97% similarity thresholds were allocated to one operational taxonomic unit (OTU) using USEARCH (version 10.0) (Edgar, 2013). Taxonomy annotation of the OTUs was performed based on the Naive Bayes classifier in QIIME2 (Davis et al., 2019) using the SILVA database (Quast et al., 2013) with a confidence threshold of 70%. Alpha and beta diversity analysis was performed to identify the complexity of species diversity of each sample utilizing QIIME2 software. Distance matrices were obtained by the Bray–Curtis algorithm. Principal coordinates analysis (PCoA) was generated by the R package. Hierarchical clustering of samples was performed by an unweighted pair-group method with arithmetic mean (UPGMA) to determine the similarity in species composition among samples. One-way analysis of variance was used to compare bacterial abundance and diversity. The online platform BMK Cloud was used to analyze the sequencing data.^1^

### Statistical analysis

GraphPad Prism version 7 (La Jolla, CA, United States) was used for statistical analysis of the data. The quantitative data met the normal distribution and the homogeneity of variance, expressed as mean ± SD. One-way ANOVA was used for comparison between groups unless noted otherwise.

## Results

### Activity of ECO against planktonic bacterial cells and inhibition of VSCs production

**Figure 1:**
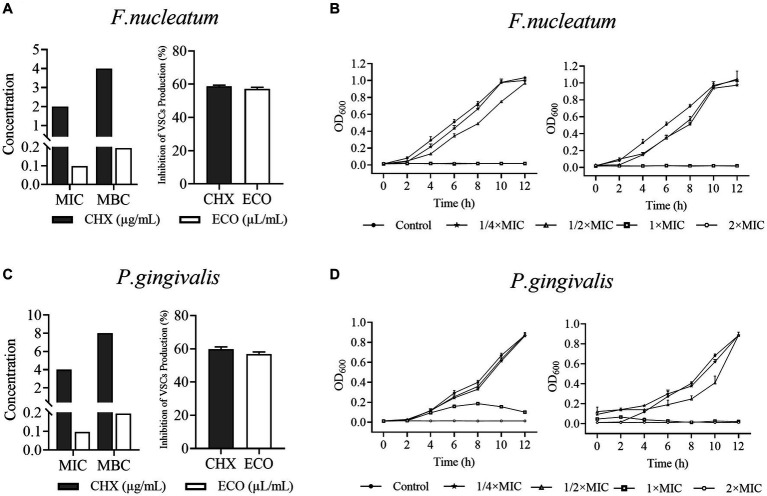
Activity of ECO against *Fusobacterium nucleatum* and *Porphyromonas gingivalis* planktonic bacterial and inhibition of their VSCs production. **(A)** MICs and MBCs of ECO and CHX against *F. nucleatum* planktonic cells, and their inhibition of *F. nucleatum* VSCs production at 1/2× MIC. **(B)** 12 h-Growth curves of *F. nucleatum* in BHI broth at different concentrations of ECO or CHX. **(C)** MICs and MBCs of ECO and CHX against *P. gingivalis* planktonic cells, and their inhibition of *P. gingivalis* VSCs production at 1/2× MIC. **(D)** 12 h-Growth curves of *P. gingivalis* in BHI broth at different concentrations of ECO or CHX.

The *in vitro* inhibitory and killing activity of ECO against planktonic cells of *F. nucleatum* and *P. gingivalis* was determined using the serial dilution method. As shown in Figures 1A,C, the MIC and MBC values of ECO for both bacteria were similar, indicating that its antibacterial activity is mainly bactericidal rather than bacteriostatic. Consistent with this observation, ECO at concentrations higher than the MIC significantly inhibited bacterial proliferation in the liquid medium, resulting in growth curves similar to those of the CHX-treated group (Figures 1B,D). When the ECO concentration was lower than the MIC, the growth of both bacteria was similar to that of the untreated group. Meanwhile, ECO showed slightly better bactericidal and growth-inhibiting activity against planktonic cells of both bacteria than CHX, demonstrating its good potential in applications. It is worth noting that since ECO is a liquid sample, the concentration units of its MIC and MBC are different from CHX. However, after performing a density conversion, the same conclusion can still be obtained. For example, the MIC of ECO against *F. nucleatum* is 0.1 μL/mL, which is approximately 2.0 μg/mL after conversion, similar to the MIC value of CHX. At sub-lethal concentrations, i.e.,1/2× MIC, ECO and CHX did not affect the growth of both bacteria (Figures 1B,D), while significantly reducing their VSCs production by approximately 60% during the anaerobic liquid culture process (Figures 1A,C). This result indicates that even if ECO does not have a killing effect on the cells of both bacteria, it can significantly inhibit their gas-producing metabolic processes.

### Activity of ECO against the bacterial biofilms

**Figure 2:**
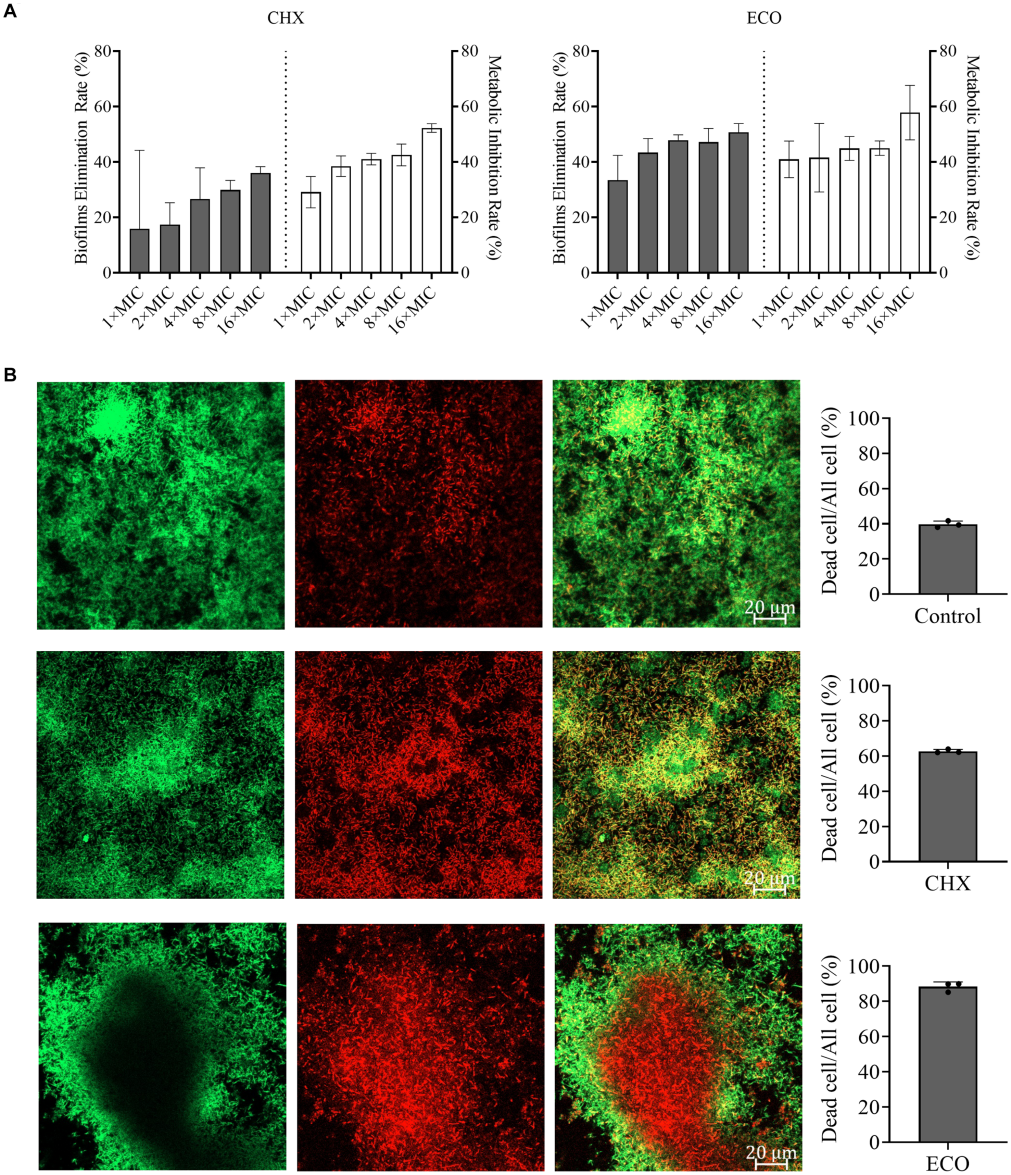
Elimination *F. nucleatum* and *P. gingivalis* mixed biofilm and inhibition of bacterial metabolism by ECO. **(A)** Determination of the biofilm elimination and metabolic inhibition by CHX (left) and ECO (right). Grey bars indicate the biofilm elimination rate and white bars indicate the metabolic inhibition rate. **(B)** CLSM observation of the mixed biofilms stained by the combination of SYTO9 (green) and PI (red) with or without the treatment (left, bar indicates 0.2 μm in the merged images). In the right panel, grey columns show the ratio of dead cells in the corresponding groups.

Various bacteria can form mixed biofilms and colonize in the oral cavity, and bacteria in biofilms normally exhibit greater tolerance to the drug treatments versus their planktonic growth state (Wang et al., 2017). Therefore, we cultured mixed biofilm of *F. nucleatum* and *P. gingivalis*, and determined the effect of ECO on its elimination, metabolic inhibition, and cell-killing ability. As shown in Figure 2A, ECO exhibited a significant dose-dependent removal effect on the mature mixed biofilms, which is slightly better than that of CHX. At 1× MIC, ECO eliminated approximately 29.10% of the biofilm, and the clearance rate increased to approximately 50% at concentrations from 4× to 16× MIC. Similar to CHX, ECO showed a significant metabolic inhibitory effect on bacterial cells in the mixed biofilm, achieving a close to 35% inhibition rate even at a lower concentration (1× MIC), which was consistent with the results obtained in planktonic cells as mentioned above.

To investigate the killing effect of ECO on bacterial cells in the biofilm, CLSM was employed to observe the biofilms visualized by live-dead staining assay after drug treatment. According to the biofilm elimination results (Figure 2A), a higher concentration (4× MIC) of CHX or ECO was used in the experiment. As shown in Figure 2B, compared with the untreated group, both CHX and ECO treatments remarkably increased the red fluorescence signal and decreased the green signal in the mixed biofilm, indicating a relatively significant increase in the number of dead cells in the biofilm due to a definitive bactericidal activity. By calculating the ratio of dead cells, at a concentration of 4× MIC, ECO had a relatively superior killing effect on bacterial cells within biofilm compared to CHX. Moreover, this result also confirmed that *F. nucleatum* and *P. gingivalis* cells in the mixed biofilm exhibit significantly increased resistance to various drugs compared to their planktonic growth state.

### Antibacterial activity of major chemical components in ECO

**Figure 3:**
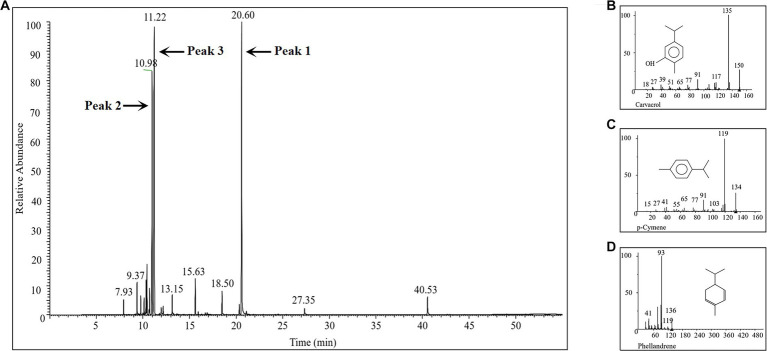
GC-MS analysis of the chemical composition of the prepared ECO sample. **(A)** TIC of the GC-MS. **(B)** Mass spectrum of peak 1 in panel **(A)**, which was identified as carvacrol. **(C)** Mass spectrum of peak 2 in panel **(A)**, which was identified as p-cymene. **(D)** Mass spectrum of peak 3 in panel **(A)**, which was identified as phellandrene.

The chemical composition of the prepared ECO was analyzed using GC-MS. A total of 137 distinct peaks were identified in the obtained total ion chromatogram (TIC, Figure 3A and Supplementary Table S1), with the highest peak intensities of the main components concentrated between 9–21 min of retention time. The relative percentage content of each peak was calculated using the area normalization method (Supplementary Table S1). By searching the MS database and matching with standard spectra, a total of 28 compounds were identified, accounting for 97.10% of the total peak area. Among these compounds, carvacrol (Figure 3B), p-cymene (Figure 3C), and phellandrene (Figure 3D) were the major chemical components of ECO, accounting for 67.83% of the total peak area. These results are consistent with previous reports (Phetsang et al., 2019; Liang et al., 2021).

**Figure 4:**
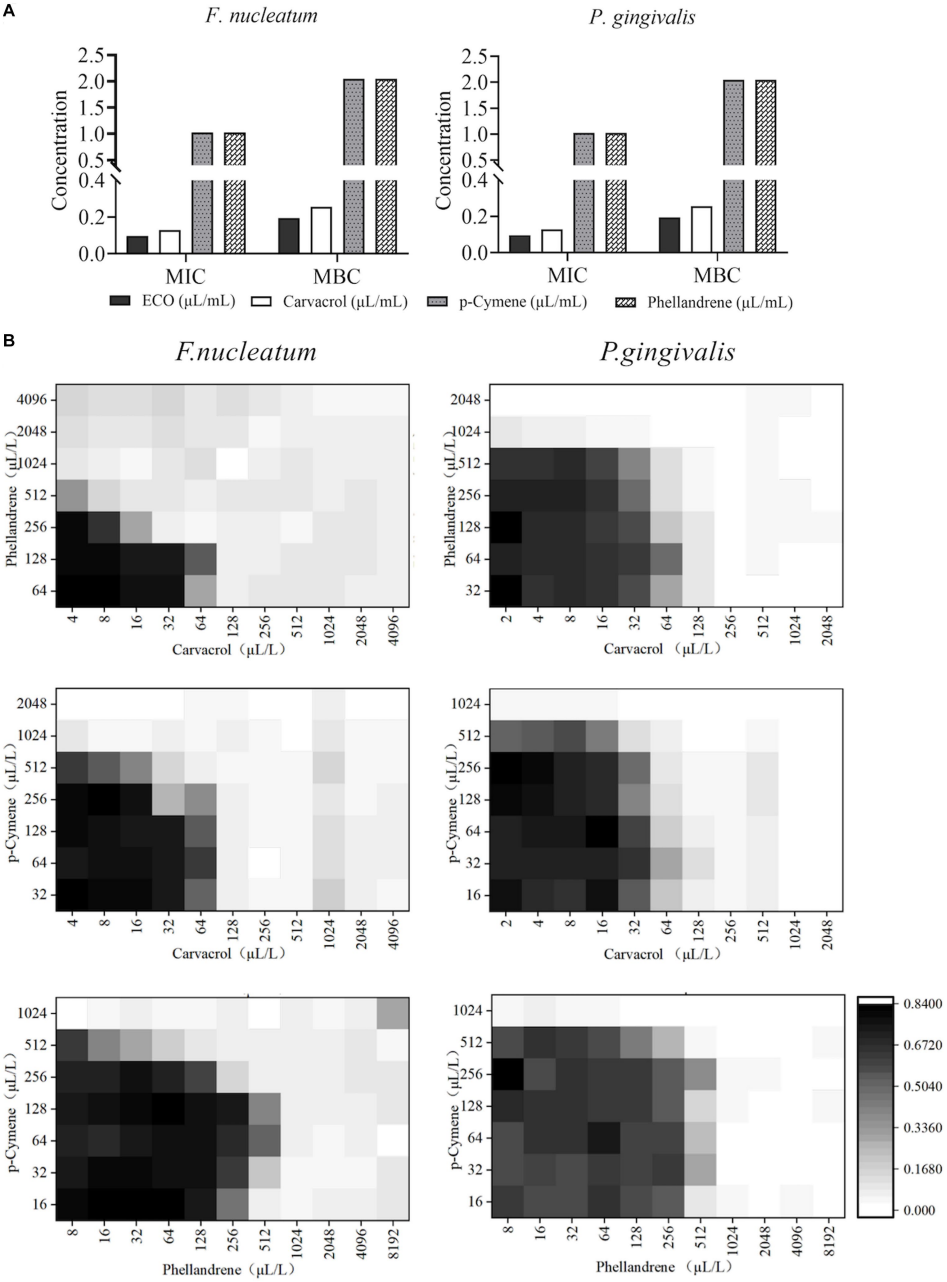
Antibacterial activity of major chemical components in ECO. **(A)** MICs and MBCs of carvacrol, p-cymene, and phellandrene against *F. nucleatum* (left) and *P. gingivalis* (right) planktonic cells. **(B)** The checkerboard results show the antibacterial effects against *F. nucleatum* (left) and *P. gingivalis* (right) by the combination of carvacrol, p-cymene, and phellandrene in pairs.

MIC and MBC results for carvacrol, p-cymene, and phellandrene showed good *in vitro* inhibitory and bactericidal activities against both *F. nucleatum* and *P. gingivalis* planktonic cells (Figure 4A). Among them, carvacrol had the best activity, with slightly bigger MIC than ECO, while the other two compounds showed significantly lower activity than ECO. Considering that carvacrol accounts for only approximately 26.25% of ECO (Supplementary Table S1), the overall activity of ECO may be derived from the cumulative effect or synergistic action of its multiple active components. Therefore, the planktonic cells of *F. nucleatum* and *P. gingivalis* were treated with the combination of the three compounds (Figure 4B), and FICI values (Table 1) were used to assess the inhibitory effects of the compound combination. The results showed that when the three compounds were combined in pairs, they exhibited certain synergistic inhibitory effects on *F. nucleatum*, and good additive inhibitory effects on *P. gingivalis*, thereby further verifying our hypothesis.

**Table 1:** Fractional inhibitory concentration index (FICI) of the combination in pairs of carvacrol, p-cymene, and phellandrene against *Fusobacterium nucleatum* and *Porphyromonas gingivalis*.

Combination	*F. nucleatum*		*P. gingivalis*	
	FICI	Evaluation	FICI	Evaluation
Carvacrol + phellandrene	0.5	Synergy	1	Additivity
Carvacrol + p-cymene	0.5	Synergy	0.75	Additivity
Phellandrene + p-cymene	0.5	Synergy	0.75	Additivity

### Effect of ECO treatment on changing the profile of VSCs

**Figure 5:**
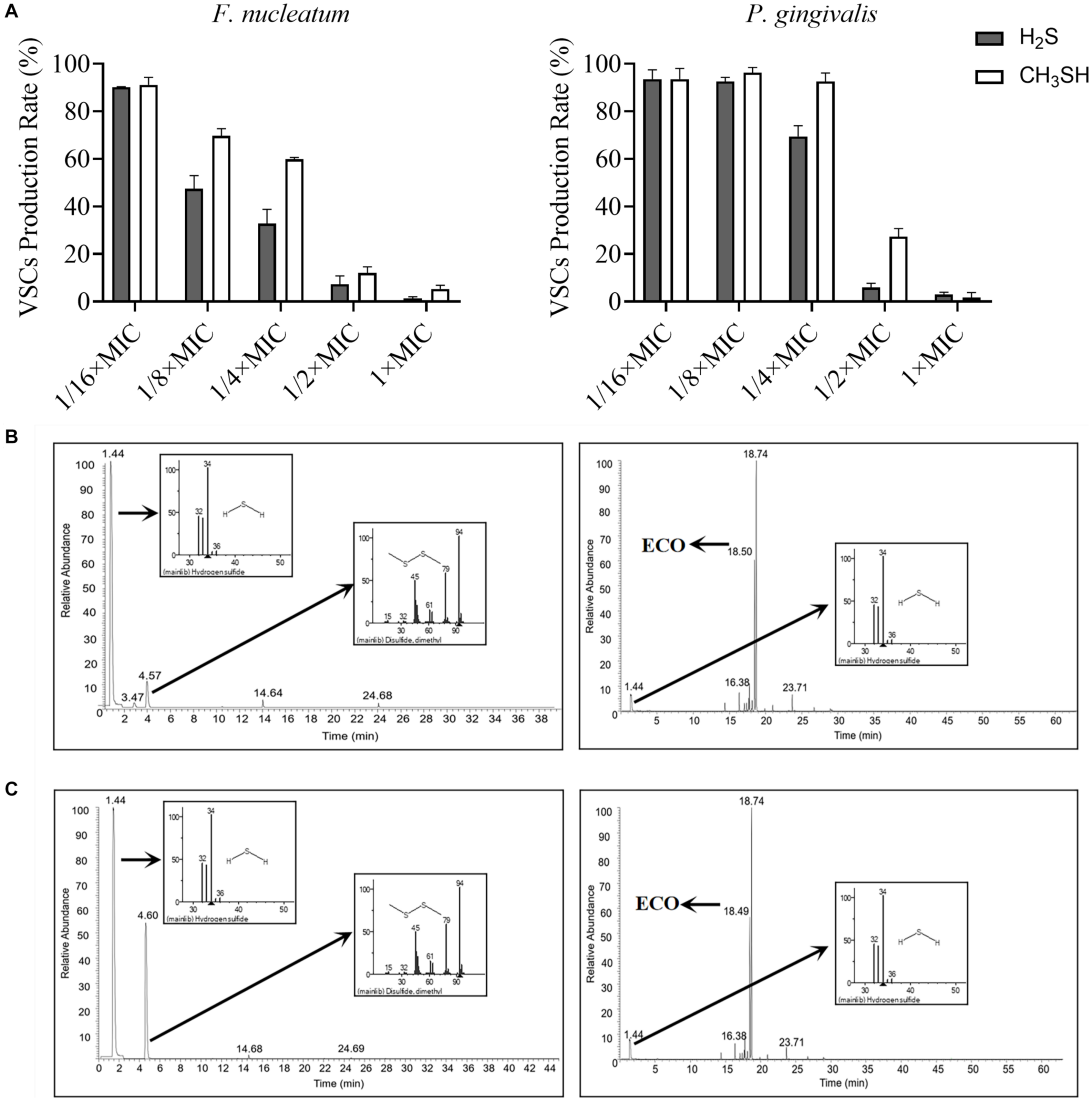
VSCs profiles produced by *F. nucleatum* and *P. gingivalis* with or without ECO treatment. **(A)** The production of hydrogen sulfide (black column) and methanethiol (white column) by *F. nucleatum* (left) and *P. gingivalis* (right) in the stimulation test. **(B)** VSCs profiles of *F. nucleatum* biofilm without (left) or with (right) the presence of 2× MIC ECO, which are shown as TICs, and the mass spectra of major peaks in the TIC are indicated in the small panels. **(C)** VSCs profiles of *P. gingivalis* biofilm without (left) or with (right) the presence of 2× MIC ECO, which are shown as TICs, and the mass spectra of major peaks in the TIC are indicated in the small panels.

Cysteine and methionine can be rapidly metabolized by oral halitosis-causing bacteria into malodorous gases such as hydrogen sulfide and methanethiol, which can trigger bad breath (Salako and Philip, 2011). Therefore, we conducted gas production stimulation tests on planktonic cells of *F. nucleatum* and *P. gingivalis* by adding L-cysteine and L-methionine in the medium. As shown in Figure 5A, the production of hydrogen sulfide and methanethiol by both bacteria was almost completely suppressed when the concentration of ECO was at the MIC level. At sub-lethal concentrations, ECO also partially inhibited the gas production process, which is consistent with our previous results (Figures 1A,C). Compared with the untreated group, ECO was more effective at inhibiting gas production in *F. nucleatum* than *P. gingivalis*, and its inhibitory effect on hydrogen sulfide production in *F. nucleatum* was the most significant.

Furthermore, the chemical composition of the gas produced during the biofilm formation of *F. nucleatum* and *P. gingivalis* was determined using GC-MS (Figures 5B,C). The major components in the gas produced by *F. nucleatum* biofilm were hydrogen sulfide, dimethyl disulfide, methanethiol, alanine ethyl ester, and methyl thioacetate, which accounted for 97.61% of the total (Supplementary Table S2). Treatment with 2× MIC ECO significantly diminished the gas production of *F. nucleatum* (Figure 5B). Except for the volatile components from ECO, only a small amount of hydrogen sulfide was detected as the main component of VSCs after the treatment, which decreased by about 92% compared to the untreated group (Supplementary Tables S2, S4). The gas produced by *P. gingivalis* biofilm was mainly composed of hydrogen sulfide, dimethyl disulfide, and methanethiol, which accounted for 96.99% of the total (Supplementary Table S3). Treatment with 2× MIC ECO significantly inhibited the gas production of *P. gingivalis* (Figure 5C). Hydrogen sulfide was also detected as the main component of VSCs, which decreased by about 76% compared to the untreated group (Supplementary Tables S3, S5).

### Effect of ECO on bacterial cell membrane integrity

**Figure 6:**
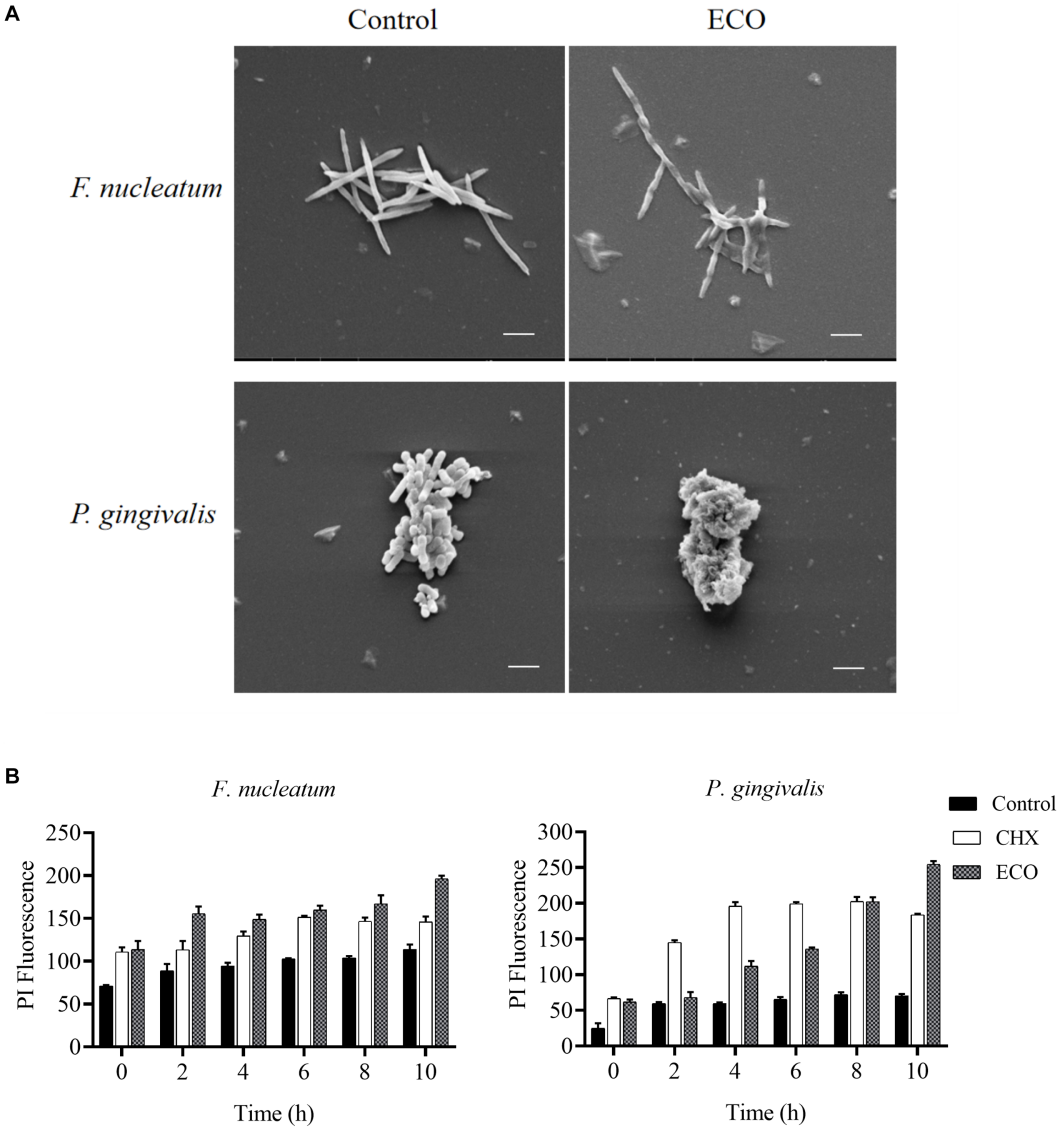
Effect of ECO on bacterial cell morphology and membrane integrity. **(A)** Effect of ECO on *F. nucleatum* (upper) and *P. gingivalis* (bottom) cell morphology observed through SEM, and bars indicate 2 μm. **(B)** Cell membrane permeability *F. nucleatum* (left) and *P. gingivalis* (right) of with or without the presence of CHX or ECO determined by PI staining.

After ECO treatment for 4 h, changes in cell morphology of *F. nucleatum* and *P. gingivalis* were examined using scanning electron microscopy (SEM). As shown in Figure 6A, untreated *F. nucleatum* cells were plump and elongated with a regular spindle shape, tapered at both ends, and slightly swollen in the middle with smooth edges. After ECO treatment, the cells exhibited fragmentation and were accompanied by ruptures and damage to the edges. Untreated *P. gingivalis* cells were short rods with smooth and plump surfaces, while after ECO treatment, the cells lost their original bacterial morphology and underwent significant fragmentation. This phenomenon is similar to the CHX treatment results reported previously (Shen et al., 2016), suggesting that ECO is likely to exert a detrimental impact on the bacterial cell membrane. This can occur either through direct interaction with the membrane or by targeting other intracellular components, ultimately resulting in cell lysis.

To further verify this hypothesis, *F. nucleatum* and *P. gingivalis* cells were stained with PI to quantitatively measure the degree of their membrane damage caused by ECO treatment, and CHX was used as a positive control. As shown in Figure 6B, compared with the untreated group, both ECO and CHX increased the cell membrane permeability of both two bacteria, and this effect became more pronounced with prolonged exposure time. Furthermore, immediate membrane damage was observed upon the addition of both CHX and ECO, suggesting that it could be attributed to the direct interaction between these compounds and the cell membrane. Overall, ECO and CHX had a stronger ability to damage the cell membrane of *F. nucleatum* than *P. gingivalis*, which is consistent with our SEM results (Figure 6A). In the shorter treatment time, the effect of ECO on *F. nucleatum* was similar to that of CHX, while CHX was more effective than ECO on *P. gingivalis*. After 10 h of treatment, the destructive effect of ECO on the cell membrane of both bacteria was significantly higher than that of CHX (*p* < 0.01).

### Effect of ECO treatment on microbial diversity of the saliva-derived bacterial biofilm

**Figure 7:**
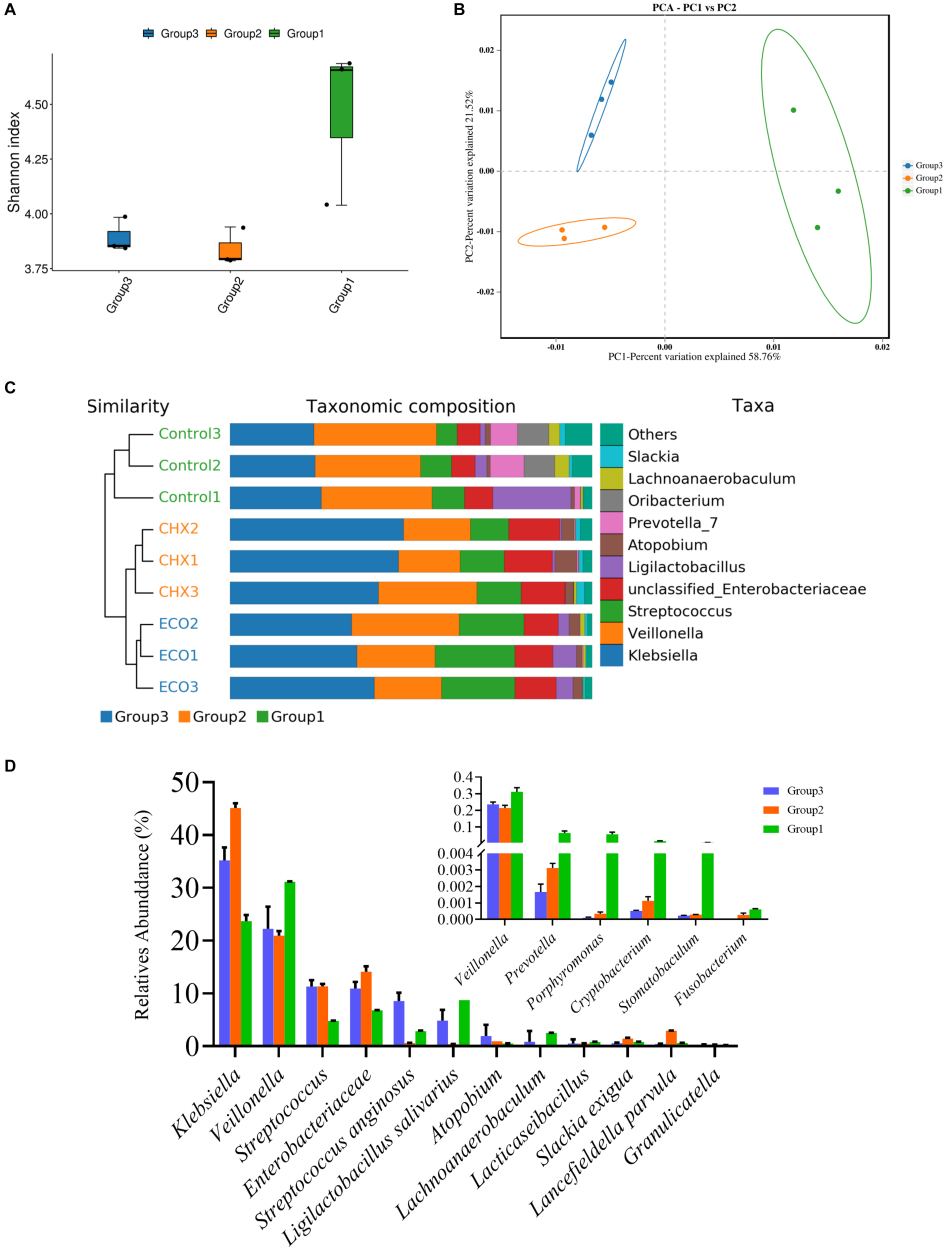
Effect of ECO treatment on microbial diversity of the saliva-derived bacterial biofilm. **(A)** Shannon’s index representing alpha-diversity of microbial communities in the saliva-derived bacterial biofilms with or without treatment. **(B)** Principal coordinates analysis (PCoA) plot based on the Bray–Curtis distance showing the relatedness of the bacterial community composition between different samples. **(C)** Combining the UPGMA clustering tree and the taxonomic composition histogram (at the genus level) shows the similarity of bacterial community structures among the different samples. **(D)** Relative abundance of main and halitosis-related (small panel) bacterial genus in different groups. In all panels, group 1 represents the untreated control group, group 2 represents the CHX-treated control group, and group 3 represents the ECO-treated control group.

The raw sequence data was processed as described above, after quality control and assembly, the clean reads from the control group were 77,049 ± 4,553, 79,694 ± 116 for the CHX group, and 79,519 ± 160 for the ECO group. The Shannon’s index was employed to access the Alpha diversity of the samples, which reflects species richness and community evenness. The index values did not have significant differences among the three groups, while a decrease was found both in CHX and ECO-treated groups compared to the control group (Figure 7A), and the index of ECO group was slightly bigger than that of CHX group. The result showed that the bacterial diversity in the untreated samples was higher than that in the treated samples. Regarding Beta diversity analysis, PCoA analysis was performed to explain the relationships between the bacterial composition of different biofilm samples, and the plot showed that the samples of three different groups were clustered separately (Figure 7B). This result revealed that microbiota in the saliva-derived bacterial biofilm with and without treatment was significantly different, and the difference in microbiota after treatment was also significant for groups CHX and ECO. It was suggested that CHX and ECO treatments altered biofilm microbiota, and the alternation was strong enough to separate the microbiota among different groups, which was confirmed by the constructed UPGMA clustering tree (Figure 7C, left panel).

At the genus level, 96.90% of the reads in all samples were classified into 9 known genera (Figure 7C, right panel). The ten most abundant genera (except the unclassified) in the control, CHX, and ECO group accounted for 94.89%, 97.35%, and 98.45%, respectively. Next, the abundance of bacteria related to oral diseases in biofilms was analyzed. As shown in Figure 7D, the average relative abundances of *F. nucleatum* than *P. gingivalis* in the untreated samples were 0.0628 ± 0.0398%, and 5.7402 ± 2.8532%, respectively; in CHX treated samples were 0.0271 ± 0.0030%, and 0.0325 ± 0.0325%, respectively; and in ECO treated samples were 0%, and 0.0103 ± 0.0010%, respectively. Similarly, bad breath-related *Prevotella*, *Porphyromonas*, *Cryptobacterium* and *Stomatobaculum* also showed a significant decrease in relative abundance in the treated samples, and the effect of ECO was slightly better than that of CHX.

## Discussion

Halitosis is a prevalent oral health issue affecting a large proportion of the population. While the condition can be caused by several factors, including poor oral hygiene, smoking, and certain medical conditions, halitosis-related bacteria in oral biofilms, such as *F. nucleatum*, *P. gingivalis*, and *Prevotella intermedia*, have been identified as a primary cause of bad breath. In addition to halitosis, *F. nucleatum* is associated with various forms of periodontal disease, including chronic gingivitis, chronic periodontitis, localized invasive periodontitis, and generalized invasive periodontitis (Tefiku et al., 2020; Chen Y. et al., 2022), as well as other health problems such as oral squamous cell carcinoma, cervical cancer, pancreatic cancer, breast cancer, and colorectal cancer (Huang et al., 2020; Parhi et al., 2020; Hong et al., 2021; McIlvanna et al., 2021; Udayasuryan et al., 2022). *P. gingivalis* infection can cause diseases such as atherosclerosis, colitis, rheumatoid arthritis, and preterm birth (Perricone et al., 2019; Tsuzuno et al., 2021; Zhang et al., 2021; Teraoka et al., 2022). *P. intermedia* is also linked to periodontal diseases and can contribute to tooth enamel degradation (Toyama et al., 2012; Jia et al., 2019). However, *P. intermedia* is a fastidious bacterium, and its cultivation is challenging and time-consuming (Horiuchi et al., 2020), which is why only *F. nucleatum* and *P. gingivalis* were used in the current study. The abundance of *Prevotella* spp. in the saliva-derived bacterial biofilm, with and without treatments, was analyzed.

Biofilms serve as the primary mode for the existence of bad breath bacteria in the oral ecosystem and significantly increase the internal bacterial resistance to various drug treatments (Eick, 2021). Therefore, in addition to planktonic cells, different bacterial biofilms, including mixed biofilms formed by *F. nucleatum* and *P. gingivalis*, and the saliva-derived bacterial biofilm, were utilized to investigate the efficacy of ECO in this study. As previously described (Rather et al., 2021), biofilms of *F. nucleatum* and *P. gingivalis* can augment the pathogenicity of both bacteria and create a favorable living environment for each other, leading to an expansion of living space and an acceleration in the development of halitosis.

In this study, multiple assays were utilized to investigate the production of VSCs by *F. nucleatum* and *P. gingivalis*. The Halimeter is a device that measures the concentration of VSCs, which is widely used in dental clinics for monitoring halitosis and evaluating treatment efficacy (Laleman et al., 2018). The rapid and convenient nature of this device makes it a suitable initial screening tool for drugs that can hinder the production of VSCs by bacterial planktonic cells. Gas production stimulation tests (Salako and Philip, 2011) were conducted to specifically evaluate the ability of *F. nucleatum* and *P. gingivalis* planktonic cells to metabolize cysteine and methionine into hydrogen sulfide and methanethiol. Additionally, GC-MS was used to determine the chemical composition of the gas produced during the biofilm formation of *F. nucleatum* and *P. gingivalis*. The results revealed that hydrogen sulfide, dimethyl disulfide, methanethiol, alanine ethyl ester, and methyl thioacetate were the major components of the gas produced by *F. nucleatum* biofilm. *P. gingivalis* biofilm mainly produced hydrogen sulfide, dimethyl disulfide, and methanethiol. Consistent with previous findings (Mark, 2021), the production of these unpleasant gases is due to the decomposition of methionine, cysteine, protein, and other nutrients in the medium by the halitosis-related bacteria.

CHX is a widely used antiseptic and disinfectant in oral health due to its broad-spectrum antimicrobial properties, low toxicity, and sustained-release effect. However, it has some side effects such as causing taste abnormalities, reversible staining of teeth, and development of resistance by microorganisms (Poppolo Deus and Ouanounou, 2022). Long-term use of CHX can also cause mucosal irritation, oral dryness, and oral mucosal desquamation (Poppolo Deus and Ouanounou, 2022). Studies suggest that the combination of CHX with other agents, such as essential oils, may enhance its efficacy and reduce its side effects (Low et al., 2014). Despite its efficacy, the use of CHX is limited by its disadvantages, making it necessary to search for alternative antimicrobial agents. Based on our results, ECO exhibited comparable activities to CHX against both planktonic cells and biofilms of *F. nucleatum* and *P. gingivalis*, and was effective in inhibiting bacterial metabolism and production of VSCs at sub-lethal concentrations. Moreover, ECO was shown to have a similar mode of action as CHX, which is to disrupt the bacterial cell membrane, causing cell leakage and interruption of metabolism, thereby inhibiting cell growth. Therefore, ECO has the potential to be used as an alternative to CHX in oral healthcare.

Essential oils are secondary metabolites of plants that possess several unique characteristics, including safety, low toxicity, distinctive aromatic scent, and volatility, and are widely used in oral healthcare products such as toothpaste, mouthwash, and oral sprays (Nordin et al., 2020; Machorowska-Pieniazek et al., 2021). While plant essential oils consist of multiple compounds, there are usually only two or three key components responsible for their antibacterial activity (Miladinovic et al., 2015; Lim et al., 2022). In the crude extracts of *E. ciliate* (Thunb.), more than 350 compounds have been identified, including monoterpenes, phenylpropanoids, flavonoids, and alkaloids (Wang et al., 2022). ECO mainly consists of small-molecule compounds such as monoterpenes, sesquiterpenes, and diterpenes (Chen S. et al., 2022). ECOs obtained from different origins, growth times, harvesting times, and extraction methods share many common chemical components, but also exhibit some differences. In this study, GC-MS was used to identify a total of 28 compounds in the prepared ECO sample, with carvacrol, p-cymene, and phellandrene being the major chemical components. After determining the MICs and MBCs of these compounds against *F. nucleatum* and *P. gingivalis* planktonic cells, they exhibited excellent *in vitro* inhibitory and bactericidal activities. Carvacrol, as a major phenolic component of ECO, has previously been shown to possess antioxidant properties (Sharifi-Rad et al., 2018), and its derivatives can act as cell membrane disruptors, leading to bactericidal effects (Oulahal and Degraeve, 2021). Furthermore, the overall activity of ECO is believed to result from the cumulative effect or synergistic action of its multiple active components, as suggested by our findings.

In recent years, there has been a growing interest in the use of natural plant products as a safer and more effective alternative to standard antimicrobial agents in the prevention and treatment of halitosis (Wylleman et al., 2021). Among various natural plant products, essential oils have gained attention due to their unique properties (Dobler et al., 2020). The present study provides experimental evidence for the potential clinical applications of ECOs in preventing and treating halitosis. Our findings reveal that ECO exhibits excellent *in vitro* inhibitory and bactericidal activities against halitosis-related bacteria, which is comparable to CHX. The results suggest that ECOs may serve as a natural alternative to conventional antimicrobial agents in oral healthcare products such as toothpaste, mouthwash, and oral sprays, thereby potentially improving oral health and preventing halitosis. However, further studies are needed to determine the efficacy and safety of ECOs in clinical settings and explore their potential applications in the development of novel oral healthcare products.

## Data availability statement

The original contributions presented in the study are included in the article/Supplementary material, further inquiries can be directed to the corresponding authors.

## Ethics statement

The studies involving human participants were reviewed and approved by Medical Ethics Committee of Qilu Hospital of Shandong University. The patients/participants provided their written informed consent to participate in this study.

## Author contributions

FL, CW, and JX conducted the experiments. FL, CW, and SP performed statistical analyses. JX, MC, SP, YX, XW, SW, and TZ provided resources and technical assistance. WH, JW, and CW provided financial support. FL, CW, and WH drafted the manuscript. WH, JW, and SP critically reviewed and revised the manuscript. All authors contributed to the article and approved the submitted version.

## Funding

This research was supported by the Taishan Industrial Experts Program (Tscy20200334, to WH), Shandong Provincial Natural Science Foundation (ZR2021MC082, to JW; ZR2021QC087, to CW), National Natural Science Foundation of China (32070100, to WH), and Science and Technology Planning Project of Traditional Chinese Medicine of Shandong Province (2019-0027, to JW).

## Conflict of interest

JX was employed by Shenzhen RELX Technology Company Limited. MC, SW, and TZ were employed by Shandong Aobo Biotechnology Company Limited. YX was employed by Beijing Xinyue Technology Company Limited.

The remaining authors declare that the research was conducted in the absence of any commercial or financial relationships that could be construed as a potential conflict of interest.

## Publisher’s note

All claims expressed in this article are solely those of the authors and do not necessarily represent those of their affiliated organizations, or those of the publisher, the editors and the reviewers. Any product that may be evaluated in this article, or claim that may be made by its manufacturer, is not guaranteed or endorsed by the publisher.

## Footnotes

^1^ https://www.biocloud.net
