# Supplementary material for: Evaluation of the antibacterial activity of Elsholtzia ciliate essential oil against halitosis-related Fusobacterium nucleatum and Porphyromonas gingivalis
Source: Front Microbiol. 2023 Aug 7;14:1219004. doi: 10.3389/fmicb.2023.1219004 (PMC10440386; doi:10.3389/fmicb.2023.1219004)
Supplement: Supplementary file 1 [file Table_1.DOCX]

**Evaluation of the Antibacterial Activity of *Elsholtzia ciliate* Essential Oil against Halitosis-related *Fusobacterium nucleatum* and *Porphyromonas gingivalis***

Fengjiao Li^1^, Chuandong Wang^2^, Jing Xu^3^, Xiaoyu Wang^2^, Meng Cao^4^, Shuhua Wang^4^, Tingting Zhang^4^, Yanyong Xu^5^, Jing Wang^1*^, Shaobin Pan^1*^, and Wei Hu^2*^

1. College of Pharmaceutical Science, Shandong University of Traditional Chinese Medicine, Jinan, Shandong 250355, China

2. State Key Laboratory of Microbial Technology, Microbial Technology Institute, Shandong University, Qingdao, Shandong 266237, China

3. Shenzhen RELX Technology Co., Ltd, Shenzhen, Guanzhou 518000, China

4. Shandong Aobo Biotechnology Co., Ltd, Liaocheng, Shandong 252800, China

5. Beijing Xinyue Technology Co., Ltd, Beijing 100020, China

*Corresponding author: hw_1@sdu.edu.cn (Wei Hu), 60030020@sdutcm.edu.cn (Jing Wang), and 60030097@sdutcm.edu.cn (Shaobin Pan)

**Table S1. GC-MS analysis results of the chemical composition of the prepared ECO sample**

| **Component Name** | **Retention Time** | **Reference m/z** | **Area** | **CAS No.** | **RSI** | **HRF Score** | **Content%** |
| --- | --- | --- | --- | --- | --- | --- | --- |
| Carvacrol | 20.597 | 135.08049 | 23488153249 | 499-75-2 | 836 | 98.3725 | 26.2465% |
| p-Cymene | 10.984 | 119.085579 | 19248268303 | 99-87-6 | 920 | 98.2384 | 21.5087% |
| Phellandrene | 11.217 | 93.069969 | 17963415697 | 99-83-2 | 781 | 97.7849 | 20.0729% |
| Spiro[2.4]heptane, 1,5-dimethyl-6-methylene- | 11.195 | 79.054321 | 7301142339 | 62238-24-8 | 610 | 99.0289 | 8.1585% |
| 1-Pentyne | 11.177 | 67.054276 | 3799799247 | 627-19-0 | 875 | 91.0092 | 4.2460% |
| Dibutyl phthalate | 40.537 | 149.023422 | 3166585788 | 84-74-2 | 956 | 96.3792 | 3.5385% |
| 3-Carene | 10.44 | 91.054321 | 1505428577 | 13466-78-9 | 938 | 98.6423 | 1.6822% |
| alpha-Pinene | 10.341 | 91.054321 | 1394166133 | 80-56-8 | 857 | 98.26 | 1.5579% |
| beta-Pinene | 9.369 | 93.069969 | 1215920385 | 127-91-3 | 907 | 98.5685 | 1.3587% |
| Cyclohexanone, 5-methyl-2-(1-methylethyl)-, (2R-cis)- | 15.622 | 139.111877 | 1006203911 | 1196-31-2 | 803 | 99.4184 | 1.1244% |
| Terpinolene | 10.719 | 121.101349 | 785486090 | 586-62-9 | 926 | 98.2188 | 0.8777% |
| Phenol, 2-methyl-5-(1-methylethyl)- | 20.346 | 135.08049 | 888381004 | 499-75-2 | 851 | 98.7759 | 0.9927% |
| 1,5-Heptadiene, 2-methyl-, (E)- | 11.168 | 95.085571 | 1028098751 | 41044-63-7 | 787 | 98.3698 | 1.1488% |
| Cyclohexanone, 5-methyl-2-(1-methylethylidene)- | 18.497 | 81.069939 | 742835720 | 15932-80-6 | 821 | 99.0951 | 0.8301% |
| beta-Pinene | 9.779 | 93.069969 | 606931553 | 127-91-3 | 919 | 98.2419 | 0.6782% |
| Cyclohexene, 3-methyl-6-(1-methylethyl)- | 10.134 | 95.085571 | 570193708 | 5256-65-5 | 872 | 98.7606 | 0.6372% |
| Terpinolene | 13.146 | 121.101349 | 568607514 | 586-62-9 | 929 | 98.4344 | 0.6354% |
| alpha-Pinene | 7.934 | 91.054321 | 474667739 | 80-56-8 | 952 | 98.431 | 0.5304% |
| Phenol, 2-methyl- | 11.965 | 108.056885 | 502229386 | 95-48-7 | 911 | 98.8645 | 0.5612% |
| 5-Methyl-2,4-diisopropylphenol | 27.35 | 177.127319 | 464477925 | 40625-96-5 | 832 | 98.4776 | 0.5190% |
| alpha-Terpinene | 12.176 | 91.054321 | 267143138 | 99-86-5 | 909 | 98.4069 | 0.2985% |
| Phenol, 2-methyl-5-(1-methylethyl)- | 21.104 | 135.08049 | 484359710 | 499-75-2 | 842 | 98.8573 | 0.5412% |
| 7-Oxabicyclo[2.2.1]heptane, 1-methyl-4-(1-methylethyl)- | 10.647 | 111.080574 | 147288422 | 470-67-7 | 773 | 99.1525 | 0.1646% |
| Eucalyptol | 11.262 | 139.111877 | 116792779 | 470-82-6 | 628 | 99.5892 | 0.1305% |
| Methyl salicylate | 16.902 | 120.02066 | 174868088 | 119-36-8 | 917 | 96.88 | 0.1954% |
| Fenchone | 13.235 | 81.069939 | 113648742 | 1195-79-5 | 905 | 99.0112 | 0.1270% |
| 2,3-Dimethyl-5-(2,6,10-trimethylundecyl) furan | 11.28 | 109.064865 | 79755588 | 166773-56-4 | 822 | 98.8955 | 0.0891% |
| Cyclohexene, 1-butyl- | 10.008 | 81.069939 | 70679994 | 3282-53-9 | 767 | 98.7262 | 0.0790% |
| Cyclohexanone, 5-methyl-2-(1-methylethyl)-, cis- | 15.942 | 112.088356 | 71438398 | 491-07-6 | 808 | 99.2657 | 0.0798% |
| Bicyclo[2.2.1]heptane, 7,7-dimethyl-2-methylene- | 10.26 | 93.069969 | 54608416 | 471-84-1 | 712 | 98.4268 | 0.0610% |
| 2-Cyclohexen-1-one, 4-(1-methylethyl)- | 16.699 | 95.049232 | 68901567 | 500-02-7 | 804 | 99.136 | 0.0770% |
| beta-Ocimene | 11.767 | 91.054321 | 48065518 | 13877-91-3 | 892 | 97.3963 | 0.0537% |
| Pentandioic acid, (p-t-butylphenyl) ester | 18.596 | 135.08049 | 53202329 | 212762-88-4 | 831 | 98.5576 | 0.0595% |
| Benzene, 1-methyl-3-(1-methylethenyl)- | 13.298 | 117.069954 | 40926185 | 1124-20-5 | 883 | 98.2185 | 0.0457% |
| (2E,4E)-3,7-Dimethylocta-2,4-diene | 10.449 | 95.085571 | 34998047 | 6874-39-1 | 889 | 100 | 0.0391% |
| Bicyclo[4.1.0]heptane, 3,7,7-trimethyl- | 9.675 | 95.085571 | 36527380 | 554-59-6 | 809 | 98.7566 | 0.0408% |
| Phenol, 4-ethyl-2-methyl- | 18.614 | 121.064903 | 48076290 | 2219-73-0 | 908 | 98.1886 | 0.0537% |
| Bicyclo[4.1.0]heptane, 3,7,7-trimethyl- | 9.527 | 95.085571 | 27584949 | 554-59-6 | 802 | 98.8872 | 0.0308% |
| Cyclohexanone, 5-methyl-2-(1-methylethenyl)-, trans- | 16.316 | 109.064865 | 29198773 | 29606-79-9 | 752 | 99.1632 | 0.0326% |
| Benzene, 1-ethyl-2,4-dimethyl- | 10.808 | 119.085579 | 23358208 | 874-41-9 | 781 | 96.2428 | 0.0261% |
| Linalool | 13.65 | 93.069969 | 25092289 | 78-70-6 | 762 | 99.5623 | 0.0280% |
| p-Cymene | 11.343 | 119.085579 | 40128234 | 99-87-6 | 828 | 98.3803 | 0.0448% |
| 1-Propanone, 1-(1-cyclohexen-1-yl)- | 16.257 | 109.064865 | 19493538 | 1655-03-4 | 862 | 99.554 | 0.0218% |
| Benzene, 1,1'-(1,1,2,2-tetramethyl-1,2-ethanediyl)bis- | 9.153 | 119.085579 | 20067282 | 1889-67-4 | 692 | 99.2526 | 0.0224% |
| trans-Ascaridol glycol | 19.733 | 109.064865 | 24011720 | 21473-37-0 | 748 | 98.4773 | 0.0268% |
| Pilocarpine | 9.419 | 109.101295 | 18230856 | 92-13-7 | 585 | 99.0904 | 0.0204% |
| 2-Cyclohexen-1-one, 3-methyl-6-(1-methylethylidene)- | 21.863 | 150.103973 | 39446504 | 491-09-8 | 693 | 98.3301 | 0.0441% |
| 3-Cyclopenten-1-one, 2,2,5,5-tetramethyl- | 15.184 | 110.072716 | 17208495 | 81396-36-3 | 645 | 97.4336 | 0.0192% |
| Tricyclo[4.1.0.0(2,7)]hept-3-ene (CAS) | 8.584 | 91.054321 | 14654243 | 35618-58-7 | 979 | 93.8703 | 0.0164% |
| Benzene, (butoxymethyl)- | 17.301 | 91.054321 | 19488947 | 588-67-0 | 827 | 98.5245 | 0.0218% |
| Terpinene | 8.458 | 93.069969 | 14768770 | 99-85-4 | 687 | 96.8035 | 0.0165% |
| beta-Caryophyllene | 24.527 | 91.054321 | 15894907 | 87-44-5 | 818 | 98.8009 | 0.0178% |
| alpha-Terpineol | 17.018 | 121.101349 | 16777904 | 98-55-5 | 754 | 98.4487 | 0.0187% |
| Benzene, (butoxymethyl)- | 11.393 | 91.054321 | 27432484 | 588-67-0 | 761 | 96.8143 | 0.0307% |
| Benzeneacetic acid, phenylmethyl ester | 18.605 | 91.054321 | 22334896 | 102-16-9 | 760 | 97.7096 | 0.0250% |
| 5-Isopropyl-3,3-dimethyl-2-methylene-2,3-dihydrofuran | 12.667 | 79.054321 | 18500889 | 81250-44-4 | 634 | 98.9013 | 0.0207% |
| 1,6-Octadiene | 10.013 | 41.038422 | 11124277 | 3710-41-6 | 581 | 97.0303 | 0.0124% |
| Bicyclo[2.2.1]heptane, 1,3,3-trimethyl- | 9.117 | 95.085571 | 10647799 | 6248-88-0 | 766 | 98.8058 | 0.0119% |
| Phenol, 2-(1-methylethyl)-, methylcarbamate | 18.11 | 121.064903 | 21810323 | 2631-40-5 | 859 | 98.5976 | 0.0244% |
| 2,3-Dimethyl-5-(2,6,10-trimethylundecyl) furan | 17.857 | 109.064865 | 13799992 | 166773-56-4 | 716 | 99.4959 | 0.0154% |
| 2-Oxabicyclo[2.2.2]octan-6-ol, 1,3,3-trimethyl-, acetate | 21.266 | 111.080574 | 14600374 | 57709-95-2 | 609 | 100 | 0.0163% |
| Fenchol | 14.331 | 81.069939 | 9704689 | 1632-73-1 | 701 | 98.9161 | 0.0108% |
| 2-Cyclohex-1-one, 5,5-dimethyl-3-hydroxy-, 1,1'-methylenebis | 22.933 | 97.064903 | 10438414 | 7599-99-7 | 541 | 99.5609 | 0.0117% |
| Propanedioic acid, mononitrile, 2-[tetrahydro-4-(4-fluorophenyl)-2,2-dimethyl-4-pyranyl]-, ethyl ester | 42.668 | 109.101295 | 9838426 | 120729-52-4 | 530 | 97.6564 | 0.0110% |
| 5-Azulenemethanol, 1,2,3,4,5,6,7,8-octahydro-.alpha.,.alpha.,3,8-tetramethyl-, acetate, [3S-(3.alpha.,5.alpha.,8.alpha.)]- | 24.207 | 161.132553 | 8830428 | 134-28-1 | 726 | 99.2805 | 0.0099% |
| cis-p-Mentha-2,8-dien-1-ol | 14.945 | 109.064865 | 8945130 | 3886-78-0 | 643 | 98.6507 | 0.0100% |
| 2-Cyclohexen-1-ol, 1-methyl-4-(1-methylethenyl)-, trans- | 14.426 | 109.064865 | 8097872 | 7212-40-0 | 663 | 98.4737 | 0.0090% |
| Ethanone, 1-(2,5-dimethylphenyl)- | 18.628 | 105.069977 | 12991281 | 2142-73-6 | 913 | 94.6007 | 0.0145% |
| 4-Undecen-6-one | 21.325 | 97.064903 | 11178043 | 32064-74-7 | 692 | 95.0151 | 0.0125% |
| Citronellol | 8.264 | 67.054276 | 6425509 | 106-22-9 | 788 | 98.6401 | 0.0072% |
| 5-Methyl-2,4-diisopropylphenol | 25.142 | 177.127319 | 8869008 | 40625-96-5 | 823 | 98.0436 | 0.0099% |
| Bicyclo[2.2.1]heptan-2-ol, 1,5,5-trimethyl- | 25.399 | 109.064865 | 12950824 | 6168-62-3 | 683 | 97.1279 | 0.0145% |
| 2-Cyclohexen-1-ol, 2-methyl-5-(1-methylethenyl)-, cis- | 17.207 | 83.049225 | 7406017 | 1197-06-4 | 577 | 99.264 | 0.0083% |
| 3-Octanone | 9.459 | 43.017841 | 5953549 | 106-68-3 | 796 | 99.7935 | 0.0067% |
| Hexane, 3,3,4,4-tetrafluoro- | 19.71 | 79.054321 | 9316199 | 648-36-2 | 712 | 93.6291 | 0.0104% |
| Anethole | 17.122 | 147.080475 | 6793312 | 104-46-1 | 714 | 98.4883 | 0.0076% |
| Bis(2-ethylhexyl) phthalate | 51.061 | 149.023422 | 7596209 | 117-81-7 | 908 | 99.3802 | 0.0085% |
| Diethyl Phthalate | 29.798 | 149.023422 | 13192435 | 84-66-2 | 934 | 96.4289 | 0.0147% |
| Dihydrocarvenyl acetate (equatorial) | 12.627 | 107.085587 | 5027277 | 4294538-94-2 | 720 | 99.1942 | 0.0056% |
| Butanedioic acid, 2,3-bis(benzoyloxy)-, (2R,3R)- | 10.817 | 105.069977 | 4749276 | 2743-38-6 | 950 | 99.8813 | 0.0053% |
| 2-(1,1-Dimethylethyl)-6-(1-methylethyl)phenol | 26.745 | 177.127319 | 6237361 | 22791-95-3 | 898 | 99.1993 | 0.0070% |
| 2-Cyclohexen-1-one, 2-methyl-5-(1-methylethenyl)-, (R)- (CAS) | 18.691 | 82.041389 | 5573149 | 6485-40-1 | 593 | 99.6928 | 0.0062% |
| 1,3-Benzodioxole, 5-(2-propenyl)- (CAS) | 20.251 | 162.067627 | 5685026 | 94-59-7 | 832 | 99.1203 | 0.0064% |
| Ethyl mandelate | 20.148 | 79.054321 | 6030866 | 774-40-3 | 780 | 98.2966 | 0.0067% |
| Phenol, 4-chloro-5-methyl-2-(1-methylethyl)- | 21.625 | 169.041534 | 5847855 | 89-68-9 | 795 | 98.9463 | 0.0065% |
| cis-Muurola-4(15),5-diene | 23.136 | 105.069977 | 5221399 | 157477-72-0 | 804 | 94.9126 | 0.0058% |
| 1-Chloromethyl-3-(1,1-dimethylethoxy)benzene | 12.83 | 107.049232 | 13387553 | 51503-06-1 | 787 | 98.6078 | 0.0150% |
| 3,6-Heptanedione | 9.635 | 43.054222 | 4200545 | 1703-51-1 | 769 | 99.7611 | 0.0047% |
| Dibutyl phthalate | 39.448 | 149.023422 | 5303508 | 84-74-2 | 916 | 97.0825 | 0.0059% |
| Norbornane, 2-chloro-1,5,5-trimethyl-, exo- | 16.041 | 95.085571 | 3687028 | 1126-28-9 | 536 | 98.154 | 0.0041% |
| Podocarpa-8,11,13-triene-7.beta.,13-diol, 14-isopropyl- | 42.952 | 199.111801 | 4333042 | 24338-19-0 | 636 | 98.5984 | 0.0048% |
| (-)-Camphor | 15.306 | 95.085571 | 3303496 | 464-48-2 | 740 | 99.762 | 0.0037% |
| 5-Azulenemethanol, 1,2,3,4,5,6,7,8-octahydro-.alpha.,.alpha.,3,8-tetramethyl-, acetate, [3S-(3.alpha.,5.alpha.,8.alpha.)]- | 27.557 | 105.069977 | 5718471 | 134-28-1 | 677 | 100 | 0.0064% |
| Propanoic acid, 2-methyl-, 2-methoxy-4-(2-propenyl)phenyl ester | 22.314 | 164.083252 | 5552780 | 84604-53-5 | 780 | 99.6945 | 0.0062% |
| DL-Menthol | 16.415 | 81.069939 | 3789002 | 89-78-1 | 755 | 99.194 | 0.0042% |
| 1-Cyclohexene-1-carboxaldehyde, 2,6,6-trimethyl- | 14.819 | 137.096237 | 3093496 | 432-25-7 | 620 | 99.8193 | 0.0035% |
| Phenol, 3,5-dimethyl- | 10.386 | 122.072701 | 2897412 | 108-68-9 | 876 | 96.3693 | 0.0032% |
| Tetrahydrocarvone | 17.216 | 41.038422 | 3143921 | 499-70-7 | 626 | 99.2644 | 0.0035% |
| 1,3-Heptadiene, 3-ethyl-2-methyl- | 10.606 | 109.101295 | 3010330 | 61142-35-6 | 971 | 97.5295 | 0.0034% |
| p-Tolyl heptanoate | 12.789 | 108.056885 | 11954015 | 71662-19-6 | 799 | 99.0516 | 0.0134% |
| Styrene | 6.646 | 104.062096 | 2667173 | 100-42-5 | 889 | 98.3862 | 0.0030% |
| Furan, 2-butyltetrahydro- | 20.84 | 71.049194 | 2445994 | 1004-29-1 | 774 | 96.6866 | 0.0027% |
| 1-[(5-Chlorothiophene-2-)sulfonyl]piperazine, N-acetyl- | 20.337 | 127.075455 | 3051380 | 1002406-98-5 | 529 | 93.2335 | 0.0034% |
| 2-Cyclopenten-1-one, 3,4,5-trimethyl- | 23.931 | 109.064865 | 2760881 | 55683-21-1 | 707 | 98.2414 | 0.0031% |
| Pentanoic acid, 5-hydroxy-, 2,4-di-t-butylphenyl esters | 27.179 | 191.143112 | 2871323 | 166273-38-7 | 844 | 100 | 0.0032% |
| (1S,4R,5R)-1,3,3-Trimethyl-2-oxabicyclo[2.2.2]octan-5-yl acetate | 22.084 | 137.096237 | 2999854 | 81781-24-0 | 633 | 99.83 | 0.0034% |
| Artedouglasia oxide A | 18.132 | 125.096184 | 2308088 | 115403-96-8 | 592 | 100 | 0.0026% |
| 2,2-Dinitropropane-1,3-diyl diacetate | 14.954 | 43.017841 | 2636414 | 26329-29-3 | 619 | 94.9179 | 0.0029% |
| 2,2-Bis(4-hydroxy-3-methylphenyl)-4-methylpentane | 44.507 | 241.122559 | 3147078 | 50628-55-2 | 516 | 97.7636 | 0.0035% |
| Bis(hexahydro-7a-methyl-3-oxo-3H-pyrrolizin-5-yl)ether | 25.299 | 139.111877 | 2860334 | 122746-21-8 | 680 | 99.948 | 0.0032% |
| 2,4,7,9-Tetramethyl-5-decyn-4,7-diol | 42.776 | 109.101295 | 2690889 | 126-86-3 | 626 | 96.6355 | 0.0030% |
| 3-Hexyne-2,5-diol, 2,5-dimethyl- | 14.155 | 43.054222 | 2227514 | 142-30-3 | 626 | 98.5034 | 0.0025% |
| Phenol, 3-(1-methylethyl)- | 19.15 | 121.064903 | 3633670 | 618-45-1 | 985 | 99.5646 | 0.0041% |
| Boron, diethyl(2,4-pentanedionato)- | 12.293 | 139.111877 | 1973290 | 19469-60-4 | 756 | 100 | 0.0022% |
| 1,2,3,1',2',3'-Hexamethyl-bicyclopentyl-2,2'-diene | 16.437 | 41.038422 | 2531569 | 51999-35-0 | 576 | 95.1192 | 0.0028% |
| 1,6-Octadiene, 5,7-dimethyl-, (R)- | 7.397 | 81.069939 | 1832970 | 85006-04-8 | 754 | 98.1542 | 0.0020% |
| Isophorone | 19.561 | 82.041389 | 2226939 | 78-59-1 | 676 | 99.8834 | 0.0025% |
| Cyclohexanemethanol, .alpha.,.alpha.,4-trimethyl-, trans- | 15.414 | 81.069939 | 2120937 | 5114-00-1 | 663 | 99.841 | 0.0024% |
| Cyclopentene, 1-isopropyl-4,5-dimethyl- | 9.027 | 95.085571 | 1769614 | 7712-74-5 | 877 | 100 | 0.0020% |
| Bis-bicyclo [2.2.1] hept-2-ylketone | 42.511 | 95.049232 | 1984573 | 4294745-07-8 | 527 | 96.4116 | 0.0022% |
| Cyclopentene, 3,3'-oxybis- | 17.843 | 83.049225 | 2859619 | 15131-55-2 | 912 | 99.5386 | 0.0032% |
| 4,6-Decadiene | 9.895 | 109.101295 | 1761191 | 55682-65-0 | 797 | 99.4538 | 0.0020% |
| (+-)-Intermedeol | 21.527 | 97.064903 | 2408997 | 136777-50-9 | 783 | 97.5235 | 0.0027% |
| Menthol | 16.136 | 81.069939 | 1312413 | 89-78-1 | 555 | 99.5904 | 0.0015% |
| 15.alpha.-Hydroxyculmorin | 29.671 | 95.085571 | 2041806 | 144447-99-4 | 589 | 99.779 | 0.0023% |
| Bicyclo[3.1.1]heptan-3-one, 2-hydroxy-2,6,6-trimethyl- | 12.596 | 43.017841 | 1301450 | 10136-65-9 | 620 | 99.9077 | 0.0015% |
| 2-Hydroxy-1,2-bis(2-methoxyphenyl)ethan-1-one, acetate | 42.7 | 135.11705 | 1783328 | 73867-45-5 | 678 | 89.6948 | 0.0020% |
| 2-Cyclohexen-1-one, 2-methyl-5-(1-methylethyl)-, (S)- | 18.853 | 81.069939 | 2631093 | 499-71-8 | 648 | 99.7145 | 0.0029% |
| 2,4,7,9-Tetramethyl-5-decyn-4,7-diol | 42.013 | 109.101295 | 1603352 | 126-86-3 | 625 | 98.6 | 0.0018% |
| 5-Nonen-4-one, 6-methyl- | 16.514 | 43.054222 | 1209677 | 7036-98-8 | 621 | 98.427 | 0.0014% |
| (1S,2R,4R,7R)-4-Isopropyl-7-methyl-3,8-dioxatricyclo[5.1.0.02,4]octane | 18.024 | 97.064903 | 1172700 | 1619-26-7 | 581 | 100 | 0.0013% |
| Cyclopentane, 1,1-dimethyl- | 12.654 | 69.069946 | 1561298 | 1638-26-2 | 617 | 95.8742 | 0.0017% |
| 4-tert-Butyl-2-(5-tert-butyl-2-hydroxyphenyl)phenol | 45.784 | 283.169617 | 1386221 | 22385-96-2 | 801 | 96.7183 | 0.0015% |
| Pyrazole, 3,5-dimethyl-1-(4-fluorophenyl)aminocarbonyl- | 17.104 | 109.101295 | 1010789 | 312746-30-8 | 681 | 97.6359 | 0.0011% |
| 2,2-Bis(4-hydroxy-3-methylphenyl)-4-methylpentane | 45.229 | 213.090988 | 764735 | 50628-55-2 | 542 | 99.122 | 0.0009% |
| .sigma.9(11)-Methyltestosterone | 44.818 | 173.096207 | 584493 | 1039-17-4 | 642 | 99.388 | 0.0007% |
| 5H-Cyclopropa[3,4]benz[1,2-e]azulen-5-one, 1,1a,1b,4,4a,7a,7b,8,9,9a-decahydro-7b,9,9a-trihydroxy-3-(hydroxymethyl)-1,1,6,8-tetramethyl-, [1aR-(1a.alpha.,1b.beta.,4a.alpha.,7a.alpha.,7b.alpha.,8.alpha.,9.beta.,9a.alpha.)]- | 46.282 | 187.111771 | 408727 | 37415-57-9 | 538 | 99.7112 | 0.0005% |

**Table S2. GC-MS analysis results of VSCs profiles produced by *F. nucleatum* biofilm**

| **Component Name** | **Retention Time** | **Reference m/z** | **Area** | **CAS No.** | **RSI** | **HRF Score** | **Contant%** |
| --- | --- | --- | --- | --- | --- | --- | --- |
| Hydrogen Sulfide | 1.438 | 34.08088 | 48762242247 | 7783-06-4 | 840 | 98.6961 | 88.0009% |
| Disulfide, dimethyl | 4.582 | 93.990471 | 2752841068 | 624-92-0 | 916 | 97.5307 | 4.9680% |
| Methanethiol | 1.537 | 44.979378 | 1173387095 | 74-93-1 | 921 | 97.9961 | 2.1176% |
| dl-Alanine ethyl ester | 1.776 | 43.989265 | 611789231 | 17344-99-9 | 850 | 99.1186 | 1.1041% |
| Methyl thiolacetate | 3.472 | 43.017765 | 784992022 | 1534-08-3 | 807 | 99.7703 | 1.4167% |
| Dimethyl trisulfide | 14.639 | 125.962555 | 295415146 | 3658-80-8 | 847 | 97.4409 | 0.5331% |
| dl-Alanyl-l-alanine | 1.976 | 43.989265 | 343482703 | 59247-16-4 | 799 | 97.5074 | 0.6199% |
| Alanine | 2.375 | 43.989265 | 227143048 | 56-41-7 | 794 | 98.0342 | 0.4099% |
| dl-Alanyl-l-alanine | 2.185 | 43.989265 | 305276101 | 59247-16-4 | 712 | 99.1724 | 0.5509% |
| Tetrasulfide, dimethyl | 24.68 | 157.934555 | 98205421 | 5756-24-1 | 847 | 97.2402 | 0.1772% |
| Butanethioic acid, S-methyl ester | 11.105 | 103.021126 | 19912047 | 2432-51-1 | 752 | 99.5111 | 0.0359% |
| Hydrogen sulfide | 1.408 | 33.987122 | 9924044 | 6/4/7783 | 934 | 100 | 0.0179% |
| Acetic anhydride | 2.459 | 43.017765 | 9757366 | 108-24-7 | 866 | 98.1233 | 0.0176% |
| 4,5-Dichloro-1,3-dioxolan-2-one | 1.816 | 48.984005 | 13127087 | 3967-55-3 | 514 | 84.6883 | 0.0237% |
| p-Cymene | 17.261 | 119.085533 | 2012206 | 99-87-6 | 924 | 100 | 0.0036% |
| Decane | 18.658 | 43.054165 | 1535462 | 124-18-5 | 881 | 100 | 0.0028% |

**Table S3. GC-MS analysis results of VSCs profiles produced by *F. nucleatum* biofilm with the addition of 2×MIC ECO**

| **Component Name** | **Retention Time** | **Reference m/z** | **Area** | **CAS No.** | **RSI** | **HRF Score** | **Contant%** |
| --- | --- | --- | --- | --- | --- | --- | --- |
| p-Cymene | 18.493 | 119.085663 | 18650700128 | 99-87-6 | 954 | 99.4208 | 32.4906% |
| Phellandrene | 18.745 | 93.069946 | 16970645639 | 99-83-2 | 768 | 97.5373 | 29.5638% |
| D-Limonene | 18.731 | 79.054298 | 6785124759 | 5989-27-5 | 648 | 99.3163 | 11.8201% |
| Hydrogen Sulfide | 1.438 | 34.08088 | 3956703473 | 7783-06-4 | 999 | 98.9171 | 6.8928% |
| 3-Carene | 17.801 | 91.054291 | 1343744218 | 13466-78-9 | 932 | 98.9381 | 2.3409% |
| beta-Pinene | 16.379 | 93.069946 | 1108511880 | 127-91-3 | 919 | 98.5565 | 1.9311% |
| Bicyclo[2.2.1]heptane, 2-(1-methylpropyl)- | 18.713 | 95.085617 | 963956575 | 74663-93-7 | 589 | 98.6339 | 1.6793% |
| .alpha.-Phellandrene | 17.693 | 91.054291 | 785042489 | 99-83-2 | 831 | 98.1312 | 1.3676% |
| Carvacrol | 28.982 | 135.080597 | 650720008 | 499-75-2 | 833 | 98.9108 | 1.1336% |
| Cyclohexanone, 5-methyl-2-(1-methylethyl)-, (2R-cis)- | 23.71 | 139.111938 | 601160389 | 1196-31-2 | 790 | 99.4244 | 1.0473% |
| alpha-Pinene | 14.401 | 91.054291 | 557665785 | 80-56-8 | 944 | 98.4301 | 0.9715% |
| Terpinolene | 18.169 | 121.101311 | 497879531 | 586-62-9 | 896 | 98.0807 | 0.8673% |
| Diacetyl sulphide | 3.956 | 43.017822 | 497238847 | 3232-39-1 | 770 | 99.4902 | 0.8662% |
| 1,4-Dioxane-2,6-dione | 1.416 | 44.997169 | 475643595 | 4480-83-5 | 999 | 99.933 | 0.8286% |
| Cyclohexene, 3-methyl-6-(1-methylethyl)- | 17.41 | 95.085617 | 438322197 | 5256-65-5 | 852 | 99.1323 | 0.7636% |
| beta-Pinene | 17.086 | 93.069946 | 377020630 | 127-91-3 | 925 | 98.5326 | 0.6568% |
| (2E,4E)-3,7-Dimethylocta-2,4-diene | 18.569 | 67.054298 | 265734255 | 6874-39-1 | 803 | 99.8311 | 0.4629% |
| alpha-Terpinene | 21.01 | 121.101311 | 257104444 | 99-86-5 | 920 | 98.6513 | 0.4479% |
| Cyclohexanone, 5-methyl-2-(1-methylethylidene)- | 26.737 | 152.11972 | 154111628 | 15932-80-6 | 786 | 99.5378 | 0.2685% |
| Cyclopentane, 1-methyl-3-(2-methyl-1-propenyl)- | 17.666 | 81.069977 | 140548865 | 75873-01-7 | 775 | 99.655 | 0.2448% |
| 3-Hepten-2-one, 3-propyl- | 18.794 | 139.111938 | 135813943 | 32064-69-0 | 583 | 97.5576 | 0.2366% |
| Ethyl Acetate | 2.696 | 43.017822 | 135565335 | 141-78-6 | 847 | 99.91 | 0.2362% |
| gamma-Terpinene | 19.917 | 91.054291 | 124046422 | 99-85-4 | 862 | 98.7051 | 0.2161% |
| 7-Oxabicyclo[2.2.1]heptane, 1-methyl-4-(1-methylethyl)- | 18.093 | 111.080605 | 122879120 | 470-67-7 | 754 | 99.6402 | 0.2141% |
| p-Cymene | 18.16 | 119.085663 | 121801269 | 99-87-6 | 702 | 94.7311 | 0.2122% |
| Propanoic acid, 2-hydroxy-, methyl ester, (卤)- | 1.602 | 45.033566 | 103614133 | 547-64-8 | 913 | 100 | 0.1805% |
| Malonic acid, 2TMS | 3.699 | 147.065689 | 93662025 | 18457-04-0 | 863 | 98.5502 | 0.1632% |
| Disulfide, dimethyl | 5.353 | 93.990578 | 89471061 | 624-92-0 | 900 | 99.2228 | 0.1559% |
| BENZENE, 1-METHYL-2-(1-METHYLETHYL)- | 17.684 | 79.054298 | 84741116 | 527-84-4 | 587 | 96.9738 | 0.1476% |
| Fenchone | 21.081 | 81.069977 | 63900928 | 1195-79-5 | 814 | 98.8006 | 0.1113% |
| p-Cymene | 18.915 | 119.085663 | 57844773 | 99-87-6 | 828 | 99.6716 | 0.1008% |
| Cyclohexasiloxane, dodecamethyl- | 29.202 | 341.018005 | 55591958 | 540-97-6 | 873 | 93.8571 | 0.0968% |
| 3-Octyne, 2,2-dimethyl- | 17.248 | 81.069977 | 50757284 | 19482-57-6 | 773 | 99.5944 | 0.0884% |
| 3-Methyl-4-hexyn-3-ol | 18.798 | 43.017822 | 47635733 | 6320-68-9 | 613 | 91.0716 | 0.0830% |
| 2-Heptanone, 6-(2-furanyl)-6-methyl- | 18.938 | 109.06488 | 47323913 | 51595-87-0 | 853 | 98.5213 | 0.0824% |
| Silanol, trimethyl- | 2.239 | 75.026115 | 46551739 | 1066-40-6 | 835 | 96.4519 | 0.0811% |
| Cyclohexene, 3-methyl-6-(1-methylethyl)- | 18.444 | 95.085617 | 37774828 | 5256-65-5 | 779 | 97.4367 | 0.0658% |
| Cyclohexanone, 5-methyl-2-(1-methylethyl)-, trans- | 24.048 | 139.111938 | 37401655 | 89-80-5 | 844 | 99.3194 | 0.0652% |
| beta-Pinene | 17.59 | 43.054203 | 36823175 | 127-91-3 | 850 | 99.0418 | 0.0641% |
| Cyclopentasiloxane, decamethyl- | 23.241 | 266.999359 | 36636398 | 541-02-6 | 832 | 93.7356 | 0.0638% |
| (2E,4E)-3,7-Dimethylocta-2,4-diene | 17.935 | 67.054298 | 35278438 | 6874-39-1 | 784 | 99.2656 | 0.0615% |
| Cyclohexene, 4-methyl-1-(1-methylethyl)- | 16.824 | 95.085617 | 25298674 | 500-00-5 | 801 | 99.0352 | 0.0441% |
| Cyclopentene, 1,4-dimethyl-5-(1-methylethyl)- | 16.253 | 95.085617 | 25104368 | 61142-33-4 | 817 | 98.9701 | 0.0437% |
| p-Cymene | 16.126 | 119.085663 | 24823146 | 99-87-6 | 831 | 99.3015 | 0.0432% |
| Bicyclo[4.1.0]heptane, 3,7,7-trimethyl- | 16.558 | 95.085617 | 22785618 | 554-59-6 | 793 | 99.2043 | 0.0397% |
| beta-Ocimene | 19.502 | 91.054291 | 21395195 | 13877-91-3 | 851 | 97.0912 | 0.0373% |
| Methyl salicylate | 25.105 | 120.020721 | 21121390 | 119-36-8 | 923 | 98.395 | 0.0368% |
| Benzene, 4-ethenyl-1,2-dimethyl- | 21.194 | 117.07003 | 17054905 | 27831-13-6 | 832 | 98.9136 | 0.0297% |
| Cyclotetrasiloxane, octamethyl- | 17.108 | 281.051453 | 15807888 | 556-67-2 | 853 | 99.0026 | 0.0275% |
| Butane, 2-methyl- | 2.524 | 41.038418 | 12789448 | 78-78-4 | 763 | 100 | 0.0223% |
| Bicyclo[2.2.1]heptane, 1,3,3-trimethyl- | 16.027 | 95.085617 | 11764802 | 6248-88-0 | 742 | 99.8259 | 0.0205% |
| Allyl acetate | 2.443 | 43.017822 | 11689242 | 591-87-7 | 737 | 100 | 0.0204% |
| Ethane, 1,2-dichloro- | 3.008 | 61.991856 | 10755823 | 107-06-2 | 548 | 100 | 0.0187% |
| Benzenemethanol, .alpha.-methyl-.alpha.-(1-methyl-2-propenyl)- | 15.119 | 121.101311 | 10384566 | 61967-11-1 | 879 | 95.2172 | 0.0181% |
| 2-Heptanone, 6-(2-furanyl)-6-methyl- | 24.477 | 109.06488 | 9619787 | 51595-87-0 | 811 | 100 | 0.0168% |
| Butanethioic acid, S-methyl ester | 12.413 | 43.054203 | 9517292 | 2432-51-1 | 775 | 100 | 0.0166% |
| Ethylene glycol, TMS derivative | 2.736 | 75.026115 | 9440815 | 4403-13-8 | 754 | 100 | 0.0164% |
| Dimethyl trisulfide | 15.905 | 125.962807 | 8990997 | 3658-80-8 | 839 | 99.0507 | 0.0157% |
| Cyclohexene, 1-(1,1-dimethylethoxy)-3-methyl- | 21.826 | 97.064934 | 7419216 | 40648-24-6 | 629 | 100 | 0.0129% |
| 2-Pyridinecarboximidamide | 20.463 | 79.054298 | 7330553 | 52313-50-5 | 607 | 100 | 0.0128% |
| Cyclohexanone, 5-methyl-2-(1-methylethyl)-, trans- | 21.673 | 97.064934 | 7146106 | 89-80-5 | 609 | 99.4339 | 0.0124% |
| 1,1'-Bicyclohexyl, 4,4'-dimethyl- | 16.473 | 81.069977 | 6626492 | 54823-99-3 | 756 | 92.5501 | 0.0115% |
| Bicyclo[4.1.0]heptane, 3,7,7-trimethyl- | 15.051 | 67.054298 | 6117412 | 554-59-6 | 780 | 100 | 0.0107% |
| 4-Allyl-1,6-heptadiene-4-ol | 21.659 | 41.038418 | 5650956 | 10202-75-2 | 512 | 100 | 0.0098% |
| Bicyclo[3.1.0]hexan-2-one, 4-methyl-1-(1-methylethyl)-, (1.alpha.,4.beta.,5.alpha.)- | 24.491 | 81.069977 | 5644578 | 2506-61-8 | 616 | 98.7055 | 0.0098% |
| 2-Cyclohexen-1-one, 4-(1-methylethyl)- | 24.879 | 95.049225 | 5377539 | 500-02-7 | 739 | 100 | 0.0094% |
| Benzenemethanol, 2,4,5-trimethyl- | 26.921 | 135.080597 | 4964184 | 5/9/4393 | 736 | 100 | 0.0086% |
| Ethane, 1,2-dichloro- | 2.99 | 61.991856 | 4440797 | 107-06-2 | 774 | 100 | 0.0077% |
| 3-Pentanone, 2-methyl-4-phenyl- | 21.655 | 43.017822 | 3936885 | 20474-49-1 | 655 | 94.5915 | 0.0069% |
| Acetic acid, TMS derivative | 4.63 | 75.026115 | 3549625 | 2754-27-0 | 837 | 95.8017 | 0.0062% |
| Benzene, (3-methylbutyl)- (CAS) | 15.336 | 92.062134 | 3333403 | 2049-94-7 | 525 | 100 | 0.0058% |
| Cycloheptasiloxane, tetradecamethyl- | 34.539 | 281.051453 | 3155725 | 107-50-6 | 731 | 100 | 0.0055% |
| 1,1'-Bicycloheptyl | 17.059 | 96.09343 | 3032952 | 23183-11-1 | 602 | 97.2944 | 0.0053% |
| Fenchol | 22.328 | 81.069977 | 2425887 | 1632-73-1 | 529 | 100 | 0.0042% |
| Trifluoroacetamide, TMS derivative | 6.36 | 77.021828 | 2302132 | 55982-15-5 | 714 | 92.4277 | 0.0040% |
| (-)-trans-Caryophyllene | 32.862 | 133.10144 | 2301535 | 87-44-5 | 679 | 100 | 0.0040% |
| 3a,7-Methano-3aH-cyclopentacyclooctene, 1,4,5,6,7,8,9,9a-octahydro-1,1,7-trimethyl-, [3aR-(3a.alpha.,7.alpha.,9a.beta.)]- | 32.532 | 161.132675 | 1512779 | 469-92-1 | 724 | 100 | 0.0026% |
| 1,6-Octadiene, 5,7-dimethyl-, (R)- | 13.814 | 81.069977 | 1464684 | 85006-04-8 | 633 | 100 | 0.0026% |
| 3-Methyl-2,4-hexanedione | 16.937 | 43.054203 | 1395411 | 4220-52-4 | 724 | 100 | 0.0024% |
| Lactic acid, 2TMS | 20.102 | 149.044968 | 1376014 | 17596-96-2 | 792 | 100 | 0.0024% |
| 7-Oxabicyclo[2.2.1]heptane, 1-methyl-4-(1-methylethyl)- | 24.667 | 43.054203 | 1078662 | 470-67-7 | 599 | 100 | 0.0019% |
| Tetrahydrocarvone | 25.417 | 95.085617 | 824068 | 499-70-7 | 588 | 100 | 0.0014% |

Table S4. GC-MS analysis results of VSCs profiles produced by *P. gingivalis* biofilm

| **Component Name** | **Retention Time** | **Reference m/z** | **Area** | **CAS No.** | **RSI** | **HRF Score** | **Contant%** |
| --- | --- | --- | --- | --- | --- | --- | --- |
| Hydrogen Sulfide | 1.438 | 34.08088 | 48762242247 | 7783-06-4 | 840 | 98.6961 | 76.4087% |
| Disulfide, dimethyl | 4.62 | 93.990433 | 11513690811 | 624-92-0 | 915 | 98.4868 | 18.0416% |
| Methanethiol | 1.538 | 44.979362 | 1620563286 | 74-93-1 | 884 | 97.7239 | 2.5394% |
| 1,4-Dioxane-2,6-dione | 1.796 | 43.989281 | 412788949 | 4480-83-5 | 979 | 98.0872 | 0.6468% |
| Dimethyl trisulfide | 14.684 | 125.962532 | 112513592 | 3658-80-8 | 850 | 97.4671 | 0.1763% |
| dl-Alanyl-l-alanine | 1.996 | 43.989281 | 209237444 | 59247-16-4 | 799 | 99.2268 | 0.3279% |
| dl-Alanyl-l-alanine | 2.376 | 43.989281 | 126822148 | 59247-16-4 | 793 | 97.8756 | 0.1987% |
| dl-Alanine ethyl ester | 2.176 | 43.989281 | 239123715 | 17344-99-9 | 811 | 98.1899 | 0.3747% |
| Tetrasulfide, dimethyl | 24.695 | 157.934525 | 24782884 | 5756-24-1 | 863 | 98.2346 | 0.0388% |
| Diacetyl sulphide | 1.677 | 43.017788 | 26701346 | 3232-39-1 | 885 | 92.4702 | 0.0418% |
| Pentanethioic acid, S-propyl ester | 13.598 | 41.038399 | 8978585 | 2432-76-0 | 813 | 100 | 0.0141% |
| Butanethioic acid, S-methyl ester | 11.156 | 103.021141 | 7656949 | 2432-51-1 | 735 | 100 | 0.0120% |
| Ethyl Acetate | 2.507 | 43.017788 | 8397361 | 141-78-6 | 824 | 100 | 0.0132% |
| S-Methyl pentanethioate | 13.526 | 41.038399 | 2949802 | 42075-43-4 | 647 | 100 | 0.0046% |
| p-Cymene | 17.28 | 119.085533 | 1926876 | 99-87-6 | 924 | 100 | 0.0030% |
| Decane | 18.672 | 43.054153 | 1645808 | 124-18-5 | 885 | 100 | 0.0026% |
| Octane | 7.965 | 43.054153 | 3161231 | 111-65-9 | 897 | 100 | 0.0050% |

**Table S5. GC-MS analysis results of VSCs profiles produced by *P. gingivalis* biofilm with the addition of 2×MIC ECO**

| **Component Name** | **Retention Time** | **Reference m/z** | **Area** | **CAS No.** | **RSI** | **HRF Score** | **Contant%** |
| --- | --- | --- | --- | --- | --- | --- | --- |
| p-Cymene | 18.493 | 119.085739 | 17572356461 | 99-87-6 | 948 | 98.9384 | 25.8871% |
| Phellandrene | 18.744 | 93.070068 | 16303430731 | 99-83-2 | 803 | 98.1939 | 24.0178% |
| Hydrogen Sulfide | 1.438 | 34.08088 | 11598829407 | 57044-25-4 | 931 | 99.9986 | 17.0871% |
| 3-[(4-Pyridinylmethyl)amino]-1-propanol | 18.731 | 79.05439 | 6520762931 | 7783-06-4 | 647 | 99.0833 | 9.6062% |
| Carbon dioxide | 1.38 | 43.989353 | 4213138201 | 124-38-9 | 988 | 99.2725 | 6.2067% |
| 3-Carene | 17.795 | 91.054382 | 1141550264 | 13466-78-9 | 938 | 98.358 | 1.6817% |
| beta-Pinene | 16.373 | 93.070068 | 893063689 | 127-91-3 | 930 | 98.4041 | 1.3156% |
| .alpha.-Phellandrene | 17.688 | 91.054382 | 602652457 | 99-83-2 | 845 | 97.9129 | 0.8878% |
| Carvacrol | 28.97 | 135.080688 | 493685703 | 499-75-2 | 836 | 98.8772 | 0.7273% |
| Cyclohexanone, 5-methyl-2-(1-methylethyl)-, (2R-cis)- | 23.711 | 139.112045 | 441838786 | 1196-31-2 | 784 | 99.2264 | 0.6509% |
| alpha-Pinene | 14.4 | 91.054382 | 433666627 | 80-56-8 | 930 | 98.1703 | 0.6389% |
| Terpinolene | 18.164 | 121.101456 | 367974007 | 586-62-9 | 886 | 98.5338 | 0.5421% |
| Cyclohexene, 3-methyl-6-(1-methylethyl)- | 17.404 | 95.085716 | 335602630 | 5256-65-5 | 848 | 99.3845 | 0.4944% |
| Disulfide, dimethyl | 5.345 | 93.990646 | 304343645 | 624-92-0 | 892 | 98.4368 | 0.4484% |
| beta-Pinene | 17.08 | 93.070068 | 284942277 | 127-91-3 | 928 | 98.7361 | 0.4198% |
| (2E,4E)-3,7-Dimethylocta-2,4-diene | 18.569 | 67.054375 | 205725356 | 6874-39-1 | 799 | 99.6164 | 0.3031% |
| alpha-Terpinene | 21.01 | 121.101456 | 196985141 | 99-86-5 | 933 | 98.7257 | 0.2902% |
| L-Lactic acid | 1.602 | 45.033588 | 118812980 | 79-33-4 | 929 | 100 | 0.1750% |
| Bis(hexahydro-7a-methyl-3-oxo-3H-pyrrolizin-5-yl)ether | 18.794 | 139.112045 | 112190223 | 122746-21-8 | 691 | 100 | 0.1653% |
| Cyclopentane, 1-methyl-3-(2-methyl-1-propenyl)- | 17.665 | 81.070068 | 109738471 | 75873-01-7 | 813 | 99.475 | 0.1617% |
| Pulegone | 26.734 | 152.119904 | 103776815 | 89-82-7 | 776 | 99.4621 | 0.1529% |
| gamma-Terpinene | 19.917 | 91.054382 | 101442740 | 99-85-4 | 895 | 98.6536 | 0.1494% |
| 7-Oxabicyclo[2.2.1]heptane, 1-methyl-4-(1-methylethyl)- | 18.092 | 111.080719 | 92154559 | 470-67-7 | 760 | 99.435 | 0.1358% |
| BENZENE, 1-METHYL-2-(1-METHYLETHYL)- | 17.679 | 79.05439 | 66260299 | 527-84-4 | 604 | 97.272 | 0.0976% |
| Ethyl Acetate | 2.696 | 43.017857 | 63140101 | 141-78-6 | 841 | 100 | 0.0930% |
| Fenchone | 21.078 | 81.070068 | 49934318 | 1195-79-5 | 819 | 99.6235 | 0.0736% |
| Disiloxane, hexamethyl- | 3.695 | 147.065781 | 43530503 | 107-46-0 | 910 | 98.3348 | 0.0641% |
| p-Cymene | 18.915 | 119.085739 | 43236764 | 99-87-6 | 868 | 99.0229 | 0.0637% |
| Cyclopentane, 1-methyl-3-(2-methyl-1-propenyl)- | 17.247 | 81.070068 | 39067546 | 75873-01-7 | 761 | 99.0564 | 0.0576% |
| 2-Heptanone, 6-(2-furanyl)-6-methyl- | 18.937 | 109.065002 | 37585600 | 51595-87-0 | 851 | 99.2035 | 0.0554% |
| tert-Butyldimethylsilanol | 2.235 | 75.026176 | 34097268 | 18173-64-3 | 872 | 91.8974 | 0.0502% |
| Bicyclo[4.1.0]heptane, 3,7,7-trimethyl- | 18.439 | 95.085716 | 30543543 | 554-59-6 | 802 | 100 | 0.0450% |
| (-)-beta-Pinene | 17.584 | 93.070068 | 27782130 | 18172-67-3 | 892 | 98.3821 | 0.0409% |
| Cyclohexanone, 5-methyl-2-(1-methylethyl)-, trans- | 24.05 | 139.112045 | 27588173 | 89-80-5 | 834 | 99.5174 | 0.0406% |
| (2E,4E)-3,7-Dimethylocta-2,4-diene | 17.935 | 67.054375 | 27261740 | 6874-39-1 | 782 | 99.3582 | 0.0402% |
| 1-(2-Aminopropoxy)-2-methoxyethane | 2.393 | 43.989353 | 23065867 | 1038338-13-4 | 810 | 99.2894 | 0.0340% |
| Bicyclo[4.1.0]heptane, 3,7,7-trimethyl- | 16.819 | 95.085716 | 18733044 | 554-59-6 | 806 | 97.8131 | 0.0276% |
| Bicyclo[2.2.1]heptane, 2-(1-buten-3-yl)- | 16.251 | 95.085716 | 17991060 | 55170-90-6 | 860 | 100 | 0.0265% |
| Bicyclo[4.1.0]heptane, 3,7,7-trimethyl- | 16.552 | 95.085716 | 17213653 | 554-59-6 | 778 | 98.6811 | 0.0254% |
| 3-Octyne, 7-methyl- | 16.386 | 95.085716 | 17177079 | 37050-06-9 | 967 | 100 | 0.0253% |
| Tricyclo[3.2.1.0(2,4)]octane, 8-methylene-, (1.alpha.,2.alpha.,4.alpha.,5.alpha.)- | 19.497 | 91.054382 | 16778894 | 38310-48-4 | 830 | 95.3567 | 0.0247% |
| Dimethyl trisulfide | 15.899 | 125.962875 | 16101498 | 3658-80-8 | 817 | 98.7897 | 0.0237% |
| Benzene, 4-ethenyl-1,2-dimethyl- | 21.195 | 117.070107 | 12822227 | 27831-13-6 | 830 | 98.9386 | 0.0189% |
| Methyl salicylate | 25.098 | 120.020828 | 9501147 | 119-36-8 | 919 | 100 | 0.0140% |
| Bicyclo[4.1.0]heptane, 3,7,7-trimethyl- | 16.026 | 95.085716 | 8706054 | 554-59-6 | 740 | 100 | 0.0128% |
| Cyclohexasiloxane, dodecamethyl- | 29.199 | 341.01825 | 7875445 | 540-97-6 | 825 | 92.6997 | 0.0116% |
| Cyclopropane, cyclopropylidene- | 17.926 | 79.05439 | 7379430 | 27567-82-4 | 804 | 94.5809 | 0.0109% |
| n-Hexane | 2.519 | 41.038479 | 6614917 | 110-54-3 | 772 | 100 | 0.0097% |
| Cyclotetrasiloxane, octamethyl- | 17.103 | 281.051636 | 6197240 | 556-67-2 | 837 | 95.0154 | 0.0091% |
| 1-(1,2,3-Trimethyl-cyclopent-2-enyl)-ethanone | 24.479 | 109.065002 | 6002582 | 70987-81-4 | 744 | 100 | 0.0088% |
| Ethylene glycol, TMS derivative | 2.732 | 75.026176 | 5299321 | 4403-13-8 | 737 | 100 | 0.0078% |
| 1,1'-Bicyclohexyl, 4,4'-dimethyl- | 16.467 | 81.070068 | 4944005 | 54823-99-3 | 775 | 98.2628 | 0.0073% |
| Cyclohexanone, 3-methyl-2-(1-methylethyl)-, cis-trans | 21.823 | 97.065025 | 4177857 | 28357-23-5 | 706 | 100 | 0.0062% |
| Cyclohexanone, 3-methyl-2-(1-methylethyl)-, cis-trans | 21.669 | 97.065025 | 3737117 | 28357-23-5 | 652 | 100 | 0.0055% |
| Perilla ketone | 24.497 | 95.049339 | 3401189 | 553-84-4 | 721 | 98.3839 | 0.0050% |
| 2-Cyclohexen-1-one, 4-(1-methylethyl)- | 24.876 | 95.049339 | 3370498 | 500-02-7 | 709 | 100 | 0.0050% |
| Bicyclo[2.2.1]heptane, 2-(1,1-dimethyl-2-propenyl)- | 15.048 | 41.038479 | 2937788 | 69219-08-5 | 651 | 95.8336 | 0.0043% |
| 1,2,3,4-Butanetetrol, 2,3-diacetate 1,4-dibenzoate, (R,S)- | 21.656 | 43.017857 | 2598255 | 74793-38-7 | 712 | 90.3973 | 0.0038% |
| (-)-trans-Caryophyllene | 32.86 | 105.070129 | 2383237 | 87-44-5 | 675 | 100 | 0.0035% |
| 2-Hexanoylfuran | 23.26 | 110.072899 | 2244507 | 14360-50-0 | 755 | 98.4799 | 0.0033% |
| Bicyclo[2.2.1]heptane, 1,3,3-trimethyl- | 15.043 | 81.070068 | 2061864 | 6248-88-0 | 671 | 95.9618 | 0.0030% |
| Propanoic acid, 2-hydroxy-, pentyl ester | 2.424 | 45.033588 | 1888005 | 6/5/6382 | 709 | 100 | 0.0028% |
| (-)-Camphor | 23.35 | 95.085716 | 1855155 | 464-48-2 | 720 | 100 | 0.0027% |
| 3-Isopropoxy-1,1,1,7,7,7-hexamethyl-3,5,5-tris(trimethylsiloxy)tetrasiloxane | 34.536 | 281.051636 | 509461 | 71579-69-6 | 588 | 100 | 0.0008% |
